# CO_2_ Hydrogenation: Na Doping Promotes CO and Hydrocarbon Formation over Ru/m-ZrO_2_ at Elevated Pressures in Gas Phase Media

**DOI:** 10.3390/nano13071155

**Published:** 2023-03-24

**Authors:** Grant Seuser, Raechel Staffel, Yagmur Hocaoglu, Gabriel F. Upton, Elijah S. Garcia, Donald C. Cronauer, A. Jeremy Kropf, Michela Martinelli, Gary Jacobs

**Affiliations:** 1 Catalyst and Aftertreatment Research and Development Group, Southwest Research Institute, 6220 Culebra Road, San Antonio, TX 78238, USA; 2 Department of Biomedical Engineering and Chemical Engineering, University of Texas at San Antonio, 1 UTSA Circle, San Antonio, TX 78249, USA; 3 Argonne National Laboratory, Lemont, IL 60439, USA; 4 Center for Applied Energy Research, University of Kentucky, 2540 Research Park Dr., Lexington, KY 40511, USA; 5 Department of Mechanical Engineering, University of Texas at San Antonio, 1 UTSA Circle, San Antonio, TX 78249, USA

**Keywords:** ruthenium, monoclinic zirconia, sodium, CO_2_ hydrogenation, reverse water-gas shift, Fischer-Tropsch synthesis

## Abstract

Sodium-promoted monoclinic zirconia supported ruthenium catalysts were tested for CO_2_ hydrogenation at 20 bar and a H_2_:CO_2_ ratio of 3:1. Although increasing sodium promotion, from 2.5% to 5% by weight, slightly decreased CO_2_ conversion (14% to 10%), it doubled the selectivity to both CO (~36% to ~71%) and chain growth products (~4% to ~8%) remarkably and reduced the methane selectivity by two-thirds (~60% to ~21%). For CO_2_ hydrogenation during in situ DRIFTS under atmospheric pressure, it was revealed that Na increases the catalyst basicity and suppresses the reactivity of Ru sites. Higher basicity facilitates CO_2_ adsorption, weakens the C–H bond of the formate intermediate promoting CO formation, and inhibits methanation occurring on ruthenium nanoparticle surfaces. The suppression of excessive hydrogenation increases the chain growth probability. Decelerated reduction during H_2_-TPR/TPR-MS and H_2_-TPR-EXAFS/XANES at the K-edge of ruthenium indicates that sodium is in contact with ruthenium. A comparison of the XANES spectra of unpromoted and Na-promoted catalysts after H_2_ reduction showed no evidence of a promoting effect involving electron charge transfer.

## 1. Introduction

There is a worldwide effort underway to find methods of recycling CO_2_, and one method is through its hydrogenation, a process that produces a substitute natural gas [1,2] or transportation fuels, either by Fischer–Tropsch synthesis (FTS) [3,4] or by methanol synthesis (used to produce alternative gasoline [5,6]). Sources of CO_2_ include power generation plants [7], quarry activities [8], biogas [9], direct air capture systems [10], and ocean water [11]. The H_2_ required to produce useful fuels from CO_2_ can be generated in a renewable manner through the electrolysis of H_2_O using electricity harvested from solar energy [12], wind power [13], or nuclear plants [14]. Although H_2_ has high level of energy per mass, because it is a gas, it possesses a low energy content per unit volume [15]. Because of this serious issue, there is an impetus to add hydrogen to carbon chains to produce liquid fuels with a high energy content as well as synthetic chemicals.

A practical route to the production of liquid fuels is to first produce CO from CO_2_ via the heterogeneously catalyzed reverse water-gas shift (RWGS) reaction [16]. Carbon monoxide is then blended with hydrogen and either fed to an FTS reactor [17], producing hydrocarbons that are further upgraded to produce premium diesel, jet fuels, lubricants, and waxes, or to a methanol synthesis reactor to produce gasoline through the MTG process [18]. In previous work [19], RWGS catalysts based on an alkali dopant, Na, and 1%Ru supported on monoclinic zirconia, were explored at a low pressure of 1 bar. In the current contribution, we examine the same catalyst compositions (albeit different batches) at a higher pressure of 20 bar. There are two reasons for this: (1) if CO_2_ is available at 20 bar, then converting it by RWGS at 20 bar is more practical, as this does not require the downstream compression of 1 atm gas; and (2) there is the possibility of generating at least some hydrocarbons during the RWGS step [20,21], as Ru is an active FTS catalyst [22]. In our previous work at 1 atm, the product selectivity could be tuned over a wide range from CH_4_ to CO, depending on the Na content (e.g., increased Na loading from 0% to 5% increased the selectivity to CO from ~3% to ~80% at an H_2_:CO_2_ ratio of 4:1, such that the CO could be used in FTS or MTG). It is of interest to determine whether increasing the pressure from 1 bar to 20 bar would have a beneficial impact on the product selectivity. The Sabatier reaction, which is favored under higher pressures, also likely occurs on Ru [23]. Although it involves the direct methanation of CO_2_, the mechanism may occur in two steps, whereby adsorbed CO (formed as metal carbonyls) is first produced prior to its subsequent methanation. It should be noted that oxygenates have also been postulated as intermediates in direct methanation.

The catalyst functions of metal (e.g., Ru, Pt), defect-laded support (e.g., ZrO_2_), and alkali (e.g., Na) were outlined in our previous work [19] and are rooted in the research of water–gas shift catalysis for the production and purification of H_2_ for fuel cell applications. The water–gas shift function (in the current context, run in reverse) is a synergy between the surfaces of metal nanoparticles and defect sites (e.g., O-vacancies or Type II bridging of the OH group at partially reduced Zr sites) on the surfaces of monoclinic zirconia [24,25]. On the other hand, the surfaces of Ru metal nanoparticles provide the CO hydrogenation function, which may involve either chain growth (depending on reactor design, media, and conditions) or methanation.

In work with Symyx, Honda Research-USA, Inc. (HRI-USA) found that the addition of light alkalis, especially sodium, to Pt/zirconia facilitates the water–gas shift [26]. In a subsequent collaboration with the Davis group at the University of Kentucky Center for Applied Energy Research (UK-CAER), the promoting effect of Na was explained by an associative mechanism in which formates and carbonates serve as intermediates. Prior research on related Rh/CeO_2_ catalysts pointed to formate carbon–hydrogen bond cleaving as the rate-determining step [27] of a formate associative mechanism, and this likely occurs at the interface between the metal and the support [28]. The HRI-USA/UK-CAER collaboration showed that Na significantly weakens the carbon–hydrogen bond of formate intermediates [29,30], accelerating the rate-limiting step of the catalysis of low-temperature WGS.

The effect of the Na dopant level on platinum/zirconia WGS catalysts was recently examined by researchers from the UK-CAER and UTSA [31]. The optimal Na loading depends on a careful balance between two functions. By increasing the Na doping levels, the formate C–H bond is weakened, and the most pronounced shift in the ν(CH) band in infrared spectroscopy occurs within a range of sodium loading (e.g., 1.8–2.5% by weight), where the band shifts remarkably from 2880 cm^−1^ (unpromoted case) to 2800 cm^−1^. As previously discussed, this effect tends to promote WGS and significantly boost CO conversion. However, further increases in Na loading (e.g., 5%Na) tend to deleteriously block a large fraction of Pt^0^ surface sites. This was also measured using infrared spectroscopy through an analysis of platinum carbonyl band intensities. Covering Pt^0^ sites with Na suppresses the activity of Pt, which is responsible for hydrogen transfer pathways (e.g., those involved in dehydrogenating formate and the formation of molecular H_2_) as well as the metal-catalyzed carbonate decomposition needed to remove CO_2_ from the surface. Adding too much Na inhibits CO_2_ desorption through (1) an increase in basicity (i.e., CO_2_ is acidic) and (2) a suppression of the activity of Pt. WGS catalytic testing using a fixed bed reactor showed that the addition of 2.5 wt.% sodium increased the conversion of CO by up to 5–6 times compared with that of unpromoted platinum/zirconia at temperatures of between 235 °C and 250 °C. Because Na facilitates C–H bond scission for the forward WGS reaction, according to the principle of microscopic reversibility, sodium is expected to promote C–H bond formation for the reverse reaction. As such, our recent research is aimed at exploring sodium-promoted metal/zirconia catalysts for CO_2_ hydrogenation.

As previously discussed, Pt was substituted for Ru, which possesses CO hydrogenation activity that is important for reactions such as methanation and FTS. The loading of ruthenium at 1% by weight was chosen due to its atomic equivalency with platinum at 2% by weight used in prior forward WGS research. The presence of Na has been found to improve the activity during WGS [32,33] or modify the selectivity of ruthenium catalysts, including an increase in the WGS activity [34], a decrease in the methanation activity [34], or an enhancement in chain growth [35] during aqueous-phase FTS and a decrease in methanation as well as an increase in chain growth during FTS over Ru/TiO_2_ catalysts [36] and a thin-film ruthenium catalyst that was supplied with sodium by electro-pumping from a solid electrolyte (Na–β″-alumina) [37]. Other elements that have been found to decrease methanation activity during CO hydrogenation over Ru and Co catalysts include Group 11 elements, such as Ag [38,39] and Au [39,40]. The addition of Au to a cobalt FTS catalyst was also found to temporarily promote WGS [41].

In the current contribution, we investigated how Na doping influences product selectivity during CO_2_ hydrogenation at a higher pressure of 20 bar. As such, we examined how an increase in Na doping inhibits CH_4_ production in favor of CO and determine whether any chain growth products are formed. In this scenario, we used gas-phase media and a fixed-bed reactor system. Prior to working at a higher pressure, we first examined the effect of the H_2_:CO_2_ ratio. In our prior work, we operated under atmospheric pressure and utilized a H_2_:CO_2_ ratio of 4:1. However, in this work, we first explored whether lowering the H_2_:CO_2_ ratio to 3:1 (i.e., the stoichiometric ratio for RWGS/Fischer–Tropsch) would increase the CO selectivity (and decrease the CH_4_ selectivity). To shed light on the mechanism, the catalysts were characterized by in situ DRIFTS of the CO_2_ hydrogenation reaction at a low pressure using these realistic H_2_:CO_2_ ratios (3:1 and 4:1 rather than 15:1, as used previously [19]). To gain insight into the question of whether CO_2_ hydrogenation occurs directly or via a CO intermediate, the catalysts were subjected to a temperature programmed reaction by preadsorbing a H_2_:CO_2_ mixture at the preferred ratio of 3:1. The most promising catalysts were further tested using a fixed bed reactor at 20 bar in gas-phase media. For the purpose of experimental control, unpromoted and sodium-promoted 1%Ru/m-zirconia catalysts were prepared using a similar method to that used in our prior investigation conducted at a low pressure [19]; the new batches were characterized by the Brunauer–Emmett–Teller (BET) method, TPR/TPR-MS, TPR-EXAFS/XANES, and TPD-MS of CO_2_. These experiments confirmed that the catalysts were similar to prior batches (see the Supplementary Information for these routine characterization studies).

## 2. Materials and Methods

Zirconia extrudates (monoclinic, Alfa Aesar, Haverhill, MA, USA) were ground and sieved to a range of particles of between 63 and 125 microns in size. A mother batch of m-zirconia supported 1%Ru was first made by using the incipient wetness impregnation (IWI) of ruthenium nitrosyl nitrate (Alfa Aesar, Haverhill, MA, USA) as the precursor. After drying under ambient conditions for 8 h, the material was placed in a muffle furnace and dried at 110 °C for an additional 8 h. The temperature was then increased to 350 °C, and the catalyst was calcined in air for 4 h. This mother batch was separated into multiple batches to dope the catalyst with Na to different loadings (0.5, 1.0, 1.8, 2.5, and 5 wt.% Na). The Na was added by IWI with sodium nitrate (Alfa Aesar, Haverhill, MA, USA) used as the salt. The material was calcined at 350 °C for 4 h.

The specific surface area and pore volume were measured using N_2_ physisorption and the BET method on a Micromeritics 3-Flex instrument (Norcross, GA, USA). Samples were degassed for 12 h at 160 °C to below 0.05 Torr.

Temperature programmed reduction (TPR) was performed using an Altamira AMI-300R (Pittsburgh, PA, USA) catalyst characterization system connected to a Hiden Analytical quadrupole mass spectrometer (MS) (Warrington, UK). As the temperature was increased from 50 to 1000 °C, 10%H_2_/Ar (San Antonio, TX, USA) flowed at a ramp rate of 10 °C/min. The MS signals recorded were H_2_, H_2_O, CO, and CO_2_. Temperature-programmed desorption (TPD) was carried out using the Altamira AMI-300R instrument (Pittsburgh, PA, USA). Each catalyst was first reduced at 300 °C using 33%H_2_/He (Airgas, San Antonio, TX, USA) flowing at 30 mL/min using a thermal ramp of 1 °C/min. After activation, the temperature was lowered to 50 °C in flowing helium (Airgas, San Antonio, TX, USA) at 30 mL/min. After that, 4%CO_2_/He (Airgas, San Antonio, TX, USA) flowing at 30 mL/min was passed through the sample for 15 min. CO_2_-TPD was performed under a flow of He (30 mL/min) to 800 °C using a thermal ramp of 10 °C/min, and the mass spectrometry signal of carbon dioxide was monitored.

H_2_ TPD was performed using the Altamira AMI-300R unit (Pittsburgh, PA, USA) connected to the Hiden mass spectrometer (Warrington, UK). Each catalyst was activated at 300 °C using 33%H_2_/Ar (Airgas, San Antonio, TX, USA) flowing at 30 mL/min using a thermal ramp of 1 °C/min. After activation, the temperature was lowered to 75 °C in the flowing H_2_/Ar mixture. After that, Ar (Airgas, San Antonio, TX, USA) flowing at 30 mL/min was passed through the sample for 45 min. H_2_-TPD was performed under a flow of Ar (30 mL/min) to 350 °C using a thermal ramp of 10 °C/min, and the mass spectrometry signal of H_2_ was also monitored. After TPD, pulse calibration was carried out by sending 5 pulses of hydrogen (Airgas, San Antonio, TX, USA) from a 500 microliter sample loop into a pure Ar (Airgas, San Antonio, TX, USA) stream flowing at 30 mL/min.

X-ray absorption spectroscopy (XAS) experiments were conducted at Beamline 10 BM, which is managed by the Materials Research Collaborative Access Team at the Advanced Photon Source at Argonne National Laboratory. In situ H_2_-TPR combined with EXAFS/XANES spectroscopies was carried out. X-ray energies were tuned using a silicon (111) monochromator. A Rh-coated mirror was used to remove undesired harmonics of the beam energy. Details about the system can be found in reference [42]. Experiments were performed on six samples concurrently using a stainless-steel cylinder with 3 mm i.d. holes for supporting up to six samples. Pinhole-free sample disks consisted of 13 to 16 mg of sample (i.e., optimized for the Ru K-edge) and ~3 mg SiO_2_. The cylinder was inserted into a clamshell furnace installed on a positioning table. Kapton ports allowed the quartz tube to be viewed. Ports were provided for flowing gases as well as the placement of a thermocouple. Samples were aligned to within 20 µm to allow for precise repeated scanning. Prior to the TPR experiment, 100 mL/min of He was flowed for at least 5 min to purge the chamber. Then, pure H_2_ with a flow rate of 100 mL/min was applied, and the temperature was increased to 300 °C under a thermal ramp of 1.0 °C/min. Scans at and near the K-edge of Ru were recorded in transmission mode, and a ruthenium metal foil was used to calibrate the energy. XANES and EXAFS spectra were processed using WinXAS [43] (Berlin, Germany). EXAFS fits were performed with the software suite (Seattle, Washington, USA) consisting of Atoms [44], as well as FEFF and FEFFIT [45] using eight averaged spectra taken at the end of the TPR run. The fitting range was 3 to 10 Å^−1^ in k-space and 1.5 Å to 3.0 Å in R-space.

To assess the effects of the H_2_:CO_2_ ratio and the Na-doping level on the surface species present, as well as the methanation activity, infrared spectroscopy was employed during CO_2_ hydrogenation. In situ DRIFTS data were recorded using a Harrick (Pleasantville, NY, USA) praying mantis apparatus coupled to a Nicolet iS-10 infrared spectrometer (Waltham, MA, USA). Samples were first activated for 1 h using a 1:1 H_2_/He mixture (200 mL/min) (Airgas, San Antonio, TX, USA) at 300 °C, and 512 scans were recorded at a resolution of 4 cm^−1^. After cooling to 50 °C in 100 mL/min of flowing He (Airgas, San Antonio, TX, USA), another spectrum was recorded. Next, a blend of either 4%CO_2_: 12%H_2_: 84%He or 4%CO_2_: 16%H_2_: 80%He (Airgas, San Antonio, TX, USA) was introduced to the reaction cell with a flow rate of 80 mL/min, and scans were recorded at 50 °C. The temperature was stepped in increments of 25 °C up to 400 °C and spectra were recorded. For the purpose of experimental control, scans in 4%CO_2_ (balance He) (Airgas, San Antonio, TX, USA) were also recorded for unpromoted and 2.5 wt.% sodium-promoted catalysts.

As with our previous work at a lower pressure [19], the principle of microscopic reversibility underpinned the current investigation. To ensure experimental control with the new batches of the catalyst, it was necessary to ensure that Na-doping facilitated the formation of formate, a proposed intermediate in the forward/reverse water–gas shift, and that it promoted the rate of forward formate decomposition in steam for forward WGS by weakening the formate C–H bond (as observed in our prior work). Unpromoted and Na-doped catalysts were treated in 200 mL/min of 1:1 H_2_:He mixture (Airgas, San Antonio, TX, USA) at 300 °C for 1 h and then cooled to 225 °C in H_2_ (Airgas, San Antonio, TX, USA). Next, 75 mL/min of He (Airgas, San Antonio, TX, USA) saturated with H_2_O (saturator temperature of 31 °C) was flowed for 8 min to ensure that defect-associated OH groups were formed [46,47,48]. Then, H_2_ (Airgas, San Antonio, TX, USA) was flowed for 15 min at 100 mL/min, and the chamber was purged in 100 mL/min He. Then, 4%CO/He (Airgas, San Antonio, TX, USA) was flowed at 50 mL/min, and the catalyst was cooled to 130 °C. A spectrum was recorded at this temperature to obtain the maximum band intensities for surface formates and Ru carbonyls. It is well known that these species are quite stable when H_2_O is not present. However, if a low concentration of H_2_O is introduced, the decomposition of formate and Ru carbonyl species can be monitored at a low temperature (e.g., 130 °C). Helium (Airgas, San Antonio, TX, USA) was flowed through a bubbler that was situated within a water bath held at 31 °C. Vapor (4.4% H_2_O, balance He) was flowed through the reaction chamber at 75 mL/min. The results of forward formate decomposition in steam are provided in the Supplementary Information section.

To shed further light on the mechanism, a temperature-programmed surface reaction with the mass spectrometry (TP-rxn/MS) of CO_2_ hydrogenation was conducted in the AMI-300R unit (Pittsburgh, PA, USA) using the Hiden mass spectrometer (Warrington, UK). Each catalyst was first reduced at 300 °C using 33%H_2_/Ar (Airgas, San Antonio, TX, USA) at a flow rate of 30 mL/min with a thermal ramp of 1 °C/min. After activation, the catalyst was purged in Ar (Airgas, San Antonio, TX, USA) flowing at 50 mL/min at 300 °C for 15 min, and then the temperature was decreased to 50 °C in Ar (Airgas, San Antonio, TX, USA) flowing at 30 mL/min. After that, 4%CO_2_/12%H_2_/balance He (Airgas, San Antonio, TX, USA) was flowed at 30 mL/min through the reaction chamber for 15 min. TP-rxn/MS was performed under Ar (Airgas, San Antonio, TX, USA) flowing at 30 mL/min to 800 °C using a thermal ramp of 10 °C/min. The mass spectrometry signals of CO and CH_4_ were monitored.

CO_2_ hydrogenation catalytic tests were conducted using two different fixed bed reactor systems, a low-pressure reactor (to explore the effect of H_2_:CO_2_ on the selectivity) and a high-pressure reactor (to favor the formation of some chain growth products). With the Universal Synthetic Gas Reactor (USGR^®^), the Ru-based catalysts were mixed with an alumina diluent in a 1:1 ratio and then screened for activity at 1 bar at 300 °C with H_2_:CO_2_ ratios of 4:1 and 3:1 at a gas hourly space velocity of 60,000 mL/g_cat_/h in 15% carbon dioxide and a 60–45% hydrogen balance in nitrogen. Gas species, including carbon dioxide, carbon monoxide, methane, ethene, propane, propene, and propane, were monitored at a frequency of 1 Hz with FTIR using a Thermo Scientific Antarias IGS utilizing a Thermo Nicolet 2-m gas cell. In a high-pressure reactor, catalysts were mixed with alumina diluent in an alumina/catalyst mass ratio of 5 to diminish the formation of hot spots. Catalysts were reduced in pure hydrogen from 25 °C to 300 °C using a thermal ramp of 10 °C/min. The reactor was operated at 300 °C with a fixed H_2_:CO_2_ ratio of 3:1 (60%H_2_, 20%CO_2_, balance nitrogen) and a space velocity of 80,000 mL/g_cat_∙h. The products were analyzed after 2 h of time on-stream through the gas chromatography of bag samples. CO_2_ conversion (*X_CO2_*, Equation (1)) and selectivity to the different carbon-containing products on a carbon basis (*S_x_*, Equation (2)) were calculated by the following equations:

(1) $$X_{CO_{2}}\left(\%\right)=\frac{F_{CO_{2},in}-F_{CO_{2},out}}{F_{CO_{2},in}}\times 100$$

(2) $$S_{X}\left(\%\right)=\frac{c_{x}n_{x}}{\sum c_{x}\cdot n_{x}}\times 100$$

where *F_in_* and *F_out_* are the reactant molar flow at the exit and entrance of the reactor, respectively; *S* is selectivity of species *x*; *c* is the number of carbon atoms in species *x*; and *n* is the number of moles of species *x*.

## 3. Results

### 3.1. Catalyst Characterization

#### 3.1.1. BET Surface Area and Porosity

BET surface area and porosity data are tabulated in Table S1. The addition of 1%Ru decreased the specific surface area from 95.4 m^2^/to 90.4 m^2^/g. This was more than expected decrease due to the addition of weight. Here, it is assumed that Ru was converted into RuO_2_ after calcination. Adding sodium (here, assumed to be sodium carbonate with varying degrees of hydration, resulting in a range of “expected surface areas”) reduced the specific surface area further, from 90.4 m^2^/g for the undoped catalyst to 44.7 m^2^/g for the catalyst with 5.0 wt.% sodium. The reduction in the specific surface area caused by the addition of sodium was greater than the change anticipated with no pore blocking (i.e., by adding weight only, which impacts the denominator). Therefore, the addition of Na results in a degree of pore blocking in zirconia, which becomes more significant at higher Na loadings. Moreover, the pore volume (calculated using the BJH method) diminishes by the addition of Ru or Na or by increasing the amount of Na. The average pore diameter was not significantly altered by the addition of Ru or Na, and all values were within the range of 9.1–10.1 nm. The slight increasing trend in the pore diameter above 1%Na may indicate preferential pore filling of narrower pores.

#### 3.1.2. Chemisorption by H_2_-TPD

H_2_ chemisorption was conducted for the 1%Ru/m-zirconia catalyst as well as the 2.5%Na and 5%Na-doped catalysts. Using the TCD signal (and confirming with the mass spectrometer signal) for H_2_, the Ru metal nanoparticles were found to be completely dispersed for the unpromoted catalyst. Using the TCD signal and assuming a near spherical morphology and an H:Ru ratio of 1:1, the average Ru^0^ diameter of the unpromoted catalyst was estimated to be 0.6 nm. Because Na (e.g., a Na species) is likely in contact with Ru surfaces (to be discussed), the average diameter could not be estimated for the Na-promoted catalysts. Nevertheless, the site capacities for the promoted catalysts with 2.5 wt.% Na and 5 wt.% Na were found to be 55% and 46%, respectively, that of the unpromoted catalyst. Moreover, as shown in Figure S1, there were three H_2_ desorption peaks labelled ***a***, ***b***, and ***c***. With an increase in Na loading, the total TCD or H_2_ MS signal was diminished. The higher temperature peaks ***b*** and ***c*** were more greatly impacted than the low temperature peak ***a***.

#### 3.1.3. Catalyst Activation

**Figure 1:**
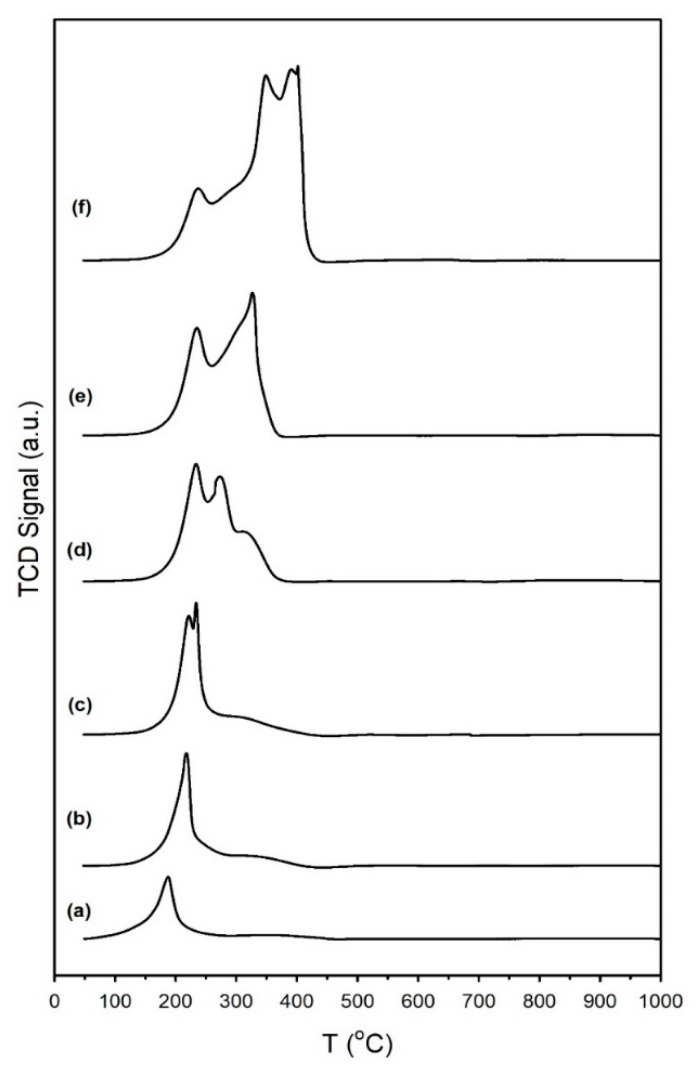
TPR profiles of (**a**) the 1%Ru/m-zirconia catalyst and the same with sodium wt. percentages of (**b**) 0.5%, (**c**) 1.0%, (**d**) 1.8%, (**e**) 2.5%, and (**f**) 5.0%.

The results of hydrogen TPR are provided in Figure 1, and the primary peak temperatures are recorded in Table 1. The results are qualitatively similar those produced in our previous work, indicating good experimental control [19]. The profiles of the unpromoted 1%Ru/m-ZrO_2_ and 0.5%Na-1%Ru/m-ZrO_2_ catalysts exhibit one sharp peak and a broad shoulder extending up to 450 °C. Increasing the loading of sodium caused the peaks to move to higher temperatures (e.g., from 187 °C for the unpromoted catalyst to between 238 °C and 402 °C for the 5%Na-doped catalyst). When the Na loading was increased above 0.5%, new peaks appeared (Table 1). The reduction process first involves the reduction of Ru oxide species to Ru metal, as corroborated by the TPR-XANES/EXAFS results. Once Ru was reduced, H_2_ easily dissociated and spilled over to the support to produce active OH groups at reduced defect sites in zirconia. In addition, O-vacancies formed with the evolution of H_2_O, as confirmed in the TPR-MS profiles of H_2_O shown in Figure S2. Finally, surface carbonates species decomposed, with the assistance of dissociated hydrogen, to CO_X_ gases, and the production of these gases increased with the Na content. Although a small signal for CO_2_ was detected in TPR-MS at Na doping loadings of 1% and higher, the predominant CO_X_ signal was that of CO, indicating the decomposition of carbonate via Ru-catalyzed decarbonylation. The addition of Na hindered the reduction processes involved during activation in H_2_. This likely indicates a direct interaction between sodium and ruthenium particles.

#### 3.1.4. Synchrotron Methods

**Figure 2:**
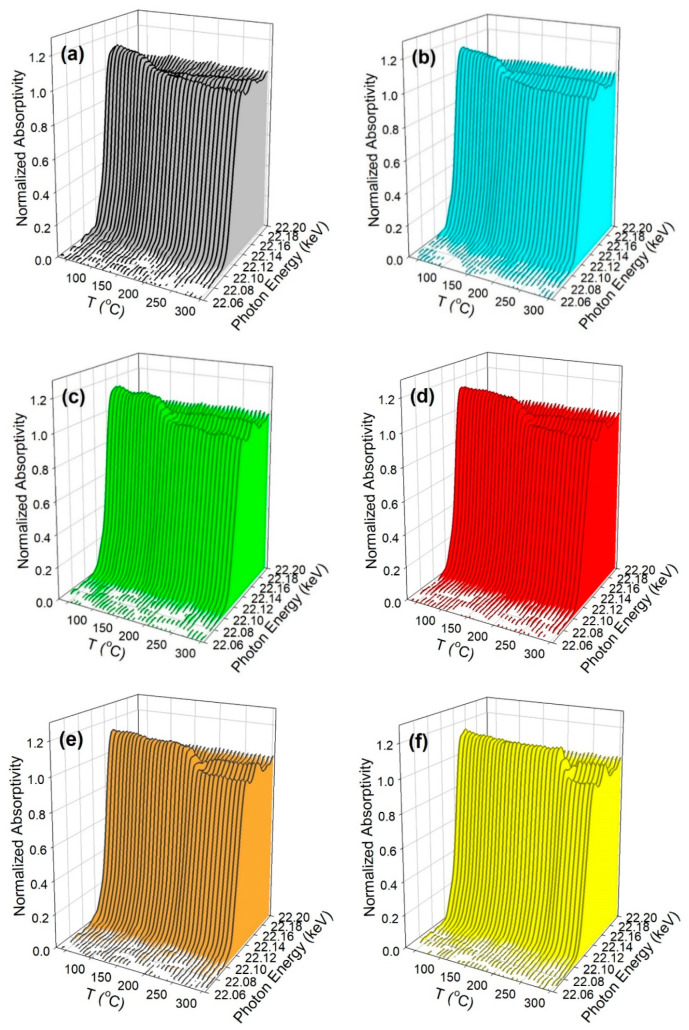
TPR-XANES waterfall plots measured at the Ru K-edge versus temperature for (**a**) 1%Ru/m-zirconia and the catalysts promoted with (**b**) 0.5 wt.% Na, (**c**) 1.0 wt.% Na, (**d**) 1.8 wt.% Na, (**e**) 2.5 wt.% Na, and (**f**) 5 wt.% Na.

The TPR-XANES spectra are provided in Figure 2, and the trends closely match those shown in our previous investigation at a low pressure [19]. In agreement with the TPR and TPR-MS profiles previously shown, increasing the Na dopant content systematically resulted in inhibited reduction. This can be most clearly observed in the linear combination fittings of the profiles with the Ru oxide and Ru^0^ reference spectra (see Supplementary Information, Figure S3). The point at which half of the Ru oxide was converted into Ru^0^ occurred at higher temperatures with the following increases in Na doping levels: no sodium, 121 °C; 0.5 wt.% Na, 129 °C; 1 wt.% Na, 149 °C; 1.8 wt.% Na, 168 °C; 2.5 wt.% Na, 203 °C; and 5 wt.% Na, 246 °C. As shown in Figure S4, there was no significant difference in the normalized XANES intensity following a reduction in H_2_, suggesting electron charge transfer [49] caused by the presence of the alkali.

TPR-EXAFS spectra provided in Figure S5 also confirm the slower reduction of Ru oxide to Ru metal nanoparticles with an increase in sodium loading. During the reduction in H_2_ with an increasing temperature, the Ru-O coordination peak centered at 1–2 Å in phase-uncorrected Fourier transform magnitude spectra decreased, while the peak for metal Ru-Ru coordination at 2–3 Å increased. As the Na doping level increased, the Ru-O coordination peak was systematically retained at higher temperatures, while the peak for Ru-Ru coordination representing the formation of Ru^0^ nanoparticles appeared at higher temperatures after the disappearance of Ru-O. For the case of unpromoted 1%Ru/m-ZrO_2_, the Ru-Ru coordination peak started to form at ~130 °C, while it began to appear at ~245 °C for the 5 wt.% Na-promoted catalyst. This inhibited reduction can likely be attributed to the partial covering of ruthenium oxide particles by Na^+^ species, as suggested in our previous work [19].

**Figure 3:**
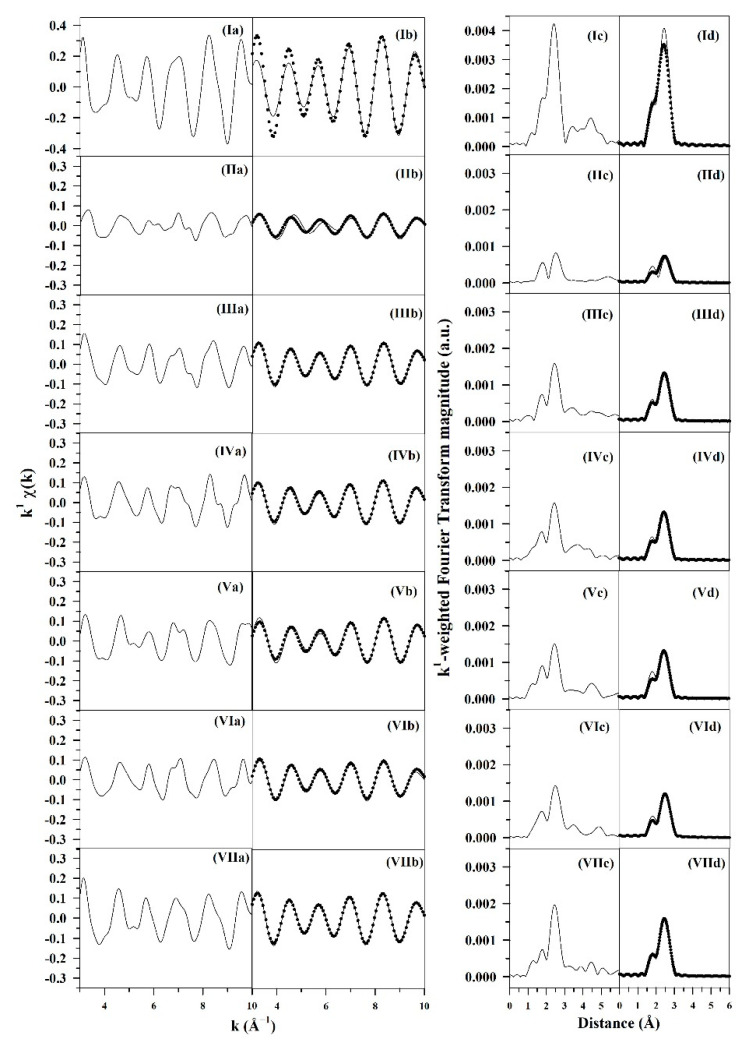
Results of fittings of the EXAFS spectra recorded at the K-edge of Ru, including the (**a**) raw k^1^-weighted χ(k) function, (**b**) (line) filtered k^1^-weighted χ(k) function and (circles) theoretical fits, (**c**) raw and (**d**) filtered k^1^-weighted Fourier transform magnitude (line) data and (circles) theoretical fits for (**I**) Ru^0^ foil, (**II**) unpromoted 1%Ru/m-zirconia, and catalysts promoted with loadings of (**III**) 0.5 wt.% Na, (**IV**) 1 wt.% Na, (**V**) 1.5 wt.% Na (**VI**) 2.5 wt.% Na, and (**VII**) 5 wt.% Na.

Theoretical fits of EXAFS spectra are compiled in Figure 3 and Table 2. The addition of Na increased the Ru-Ru coordination number from 2 for the undoped catalyst to the range of 3.1–4.5 with Na-doping. Nominally, a coordination number of 2 for the undoped 1%Ru/m-ZrO_2_ should correlate with a 3-atom cluster. The estimates provided in Table 2 range from 7 to 11 atoms for Na-doped catalysts. They were obtained by extrapolation assuming an approximately spherical cluster morphology. Using this extrapolation method, the average Ru diameter was found to be slightly smaller for the unpromoted catalyst (0.31 nm), while that of Na-doped catalysts ranged from 0.47 to 0.69 nm, and no clear trend in Ru^0^ cluster size was observed with Na loading. The average Ru cluster sizes for all catalysts were slightly smaller than those observed in our previous work [19], indicating slight variations in the Ru size between parent batches prior to Na doping. Both H_2_ chemisorption and EXAFS detected subnanometer particle sizes for the unpromoted catalyst. Because the sizes predicted by EXAFS for all catalysts should result in greater dispersion, the fact that the site capacities predicted by H_2_ chemisorption for the 2.5 wt.% Na and 5 wt.% Na catalysts were significantly lower that of the unpromoted catalyst suggests contact of a Na-containing species with Ru metal surfaces.

#### 3.1.5. Thermal Desorption of CO_2_

**Figure 4:**
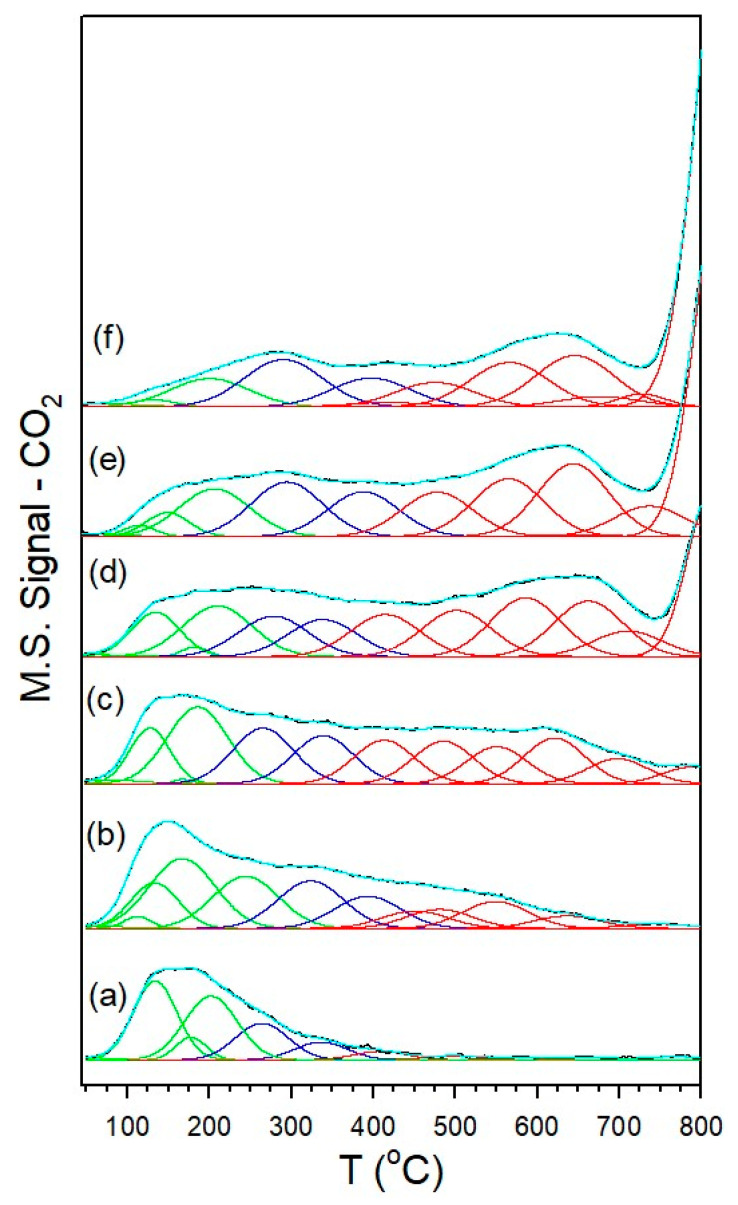
Temperature programmed desorption of CO_2_ for (**a**) 1%Ru/m-zirconia and Na-doped catalysts with loadings of (**b**) 0.5%Na, (**c**) 1%Na, (**d**) 1.8%Na, (**e**) 2.5%Na, and (**f**) 5%Na, including Gaussian fitting peaks having temperature maxima in the following ranges: (green) < 250 °C, (blue) 250 °C to 400 °C, and (red) > 400 °C (see Table 3).

TPD-MS of carbon dioxide was performed to gain insight into the impact of adding Na on the surface basicity, as CO_2_ is an acidic molecule, and its adsorption is the first step in the RWGS catalytic cycle. Figure 4 shows the TPD-MS of the CO_2_ profiles for all catalysts, including a fitting with twelve Gaussian peaks from ~50 to 800 °C. Table 3 compiles the percentages of Gaussian peaks with peak maxima located within three different temperature ranges, including below 250 °C, between 250 °C and 400 °C, and above 400 °C. The undoped 1%Ru/m-ZrO_2_ catalyst exhibited facile removal of CO_2_, as 65% of the peaks had peak maxima falling below 250 °C. With the addition of Na and with an increase in Na loading, the CO_2_ desorption peaks shifted to higher temperatures. At a dopant level of 5%Na, only 10% of the peaks had peak maxima below 250 °C, while 21% of the peaks had maxima between 250 °C and 400 °C, and 69% were positioned above 400 °C. While this may be attributed, in part, to higher catalyst basicity, the inhibition of Ru-catalyzed CO_2_ removal caused by Na blocking Ru sites cannot be ruled out as a contributor. Systematic attenuation of Ru metal activity by increasing the Na content diminished the methanation activity in favor of RWGS [19] and, in this work, this was expected to increase the probability of C–C coupling resulting in the formation of some chain growth products. As detailed in our previous work [19], increased basicity is expected to increase the strength of bonding between the O–C–O functional group of formate and the surface, thus weakening the C–H bond. Since, as previously discussed, scission of this bond is the proposed rate-determining step (RDS) of the surface formate mechanism (i.e., the associative mechanism) for the forward water–gas shift, weakening this bond could (according to the Principle of Microscopic Reversibility) aid in the formation of formate carbon–hydrogen bonds for the reverse reaction.

#### 3.1.6. In Situ Infrared Spectroscopy

Prior to conducting RWGS reactor testing, it was important to first verify that Na doping resulted in electronic modification of the formate CH bond during forward WGS, as was shown in our previous investigation at a low pressure [19]. To that end, formate was produced by reacting CO with defect-associated bridging OH groups and then subsequently decomposed in H_2_O at 130 °C. As shown in Figure S6, which shows intensities normalized by height, the position of the ν(CH) band in DRIFTS indicates that Na-doping weakened the formate bond, as there was a shift in the main formate ν(CH) band to lower wavenumbers, as follows: 0%Na, 2868 cm^−1^ (with shoulders at 2900, 2884, and 2858 cm^−1^); 0.5%Na, 2861 cm^−1^ (with shoulders at 2892, 2861, 2837, and 2834 cm^−1^); 1%Na, 2828 cm^−1^ (with a minor peak at 2853 cm^−1^ and shoulders at 2879 and 2798 cm^−1^); 1.8%Na, 2801 cm^−1^ (with a shoulder at 2850 cm^−1^); 2.5%Na, 2803 cm^−1^ (with a shoulder at 2848 cm^−1^); and 5%Na, 2815 cm^−1^ (with a shoulder at 2852 cm^−1^). Figure S7 shows that the intensity of the formate ν(CH) band at 2868 cm^−1^ was low for the undoped catalyst, as well as for loadings of 0.5%Na and 1%Na. At Na loadings of 1.8% and 2.5%, however, the formate signal was intensified. Additionally, at those loadings, formate underwent rapid forward decomposition in H_2_O. At 5%Na loading, the formate intensity was lower than that observed in the 1.8–2.5%Na range, and the formate decomposition rate in steam was slow, indicating an excessive loading of Na when forward WGS is desired. These results are consistent with those of our previous investigation [19], indicating good experimental control.

As shown in Figure S8, the addition of Na attenuated the Ru carbonyl ν(CO) bands, indicating that Na disrupts Ru^0^ sites. Using 0%Na as a reference for an Ru carbonyl band area of 100%, the area of the overall Ru carbonyl band region decreased with the addition of Na, as follows: undoped, 100%; 0.5 wt.% sodium, 65%; 1 wt.% and 1.8 wt.% sodium, 60%; 2.5 wt.% sodium, 26%; and 5 wt.% sodium, 4%. Comparing Figure S7 with Figure S9 shows that the formate ν(CH) band for the 1.8 wt.% sodium and 2.5 wt.% sodium doped catalysts (i.e., close to the optimal sodium doping level) decreased faster (i.e., via the WGS reaction) in comparison with the Ru carbonyl ν(CO) band. Although the proposed catalytic cycle remains unproven, this result favors a surface formate mechanism [27] over a support-mediated redox mechanism [50]. In the latter case, Ru-CO is proposed to react with O adatoms of m-ZrO_2_, producing CO_2_, with H_2_O replenishing O-vacancies on the surface of zirconia with O and liberating H_2_ in the process. In our previous investigation at a low pressure [19], the attenuation of the activity of the Ru metal function helped to hinder the interception of CO and the subsequent secondary reaction of methanation, improving the RWGS selectivity significantly. In the current study, in addition to this, we expect that the attenuation of the CO_X_ hydrogenation activity resulting from contact between the Na and Ru nanoparticles may have allowed for an increase in the probability of C–C coupling, resulting in the production of some chain growth products from Fischer–Tropsch synthesis.

**Figure 5:**
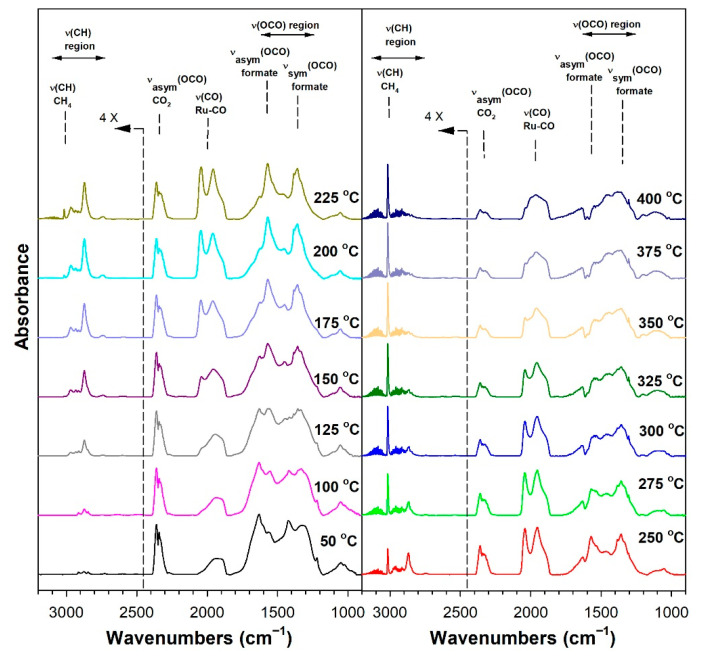
DRIFTS of CO_2_ hydrogenation with 4%CO_2_ + 12%H_2_ (balance He) over 1%Ru/m-zirconia activated at 300 °C in hydrogen, purged in helium, and then cooled to 50 °C prior to the reaction.

**Figure 6:**
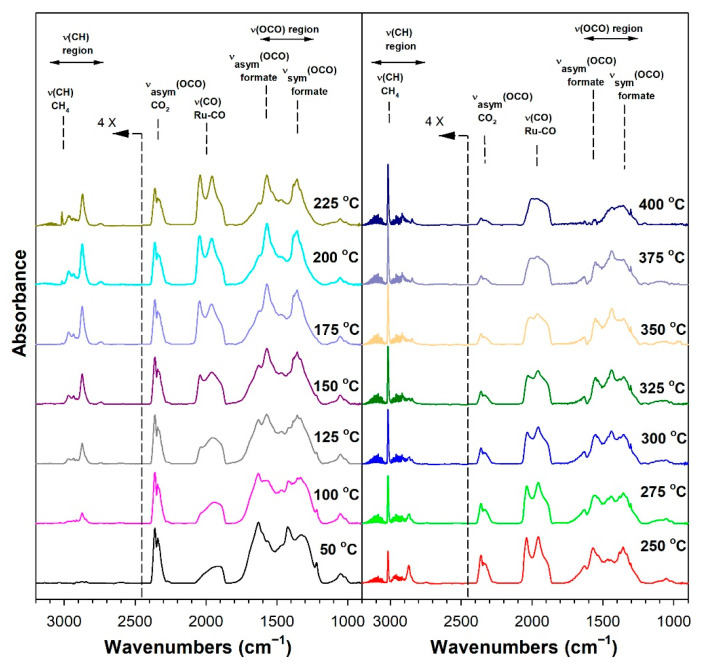
DRIFTS of CO_2_ hydrogenation with 4%CO_2_ + 16%H_2_ (balance He) over 1%Ru/m-zirconia activated at 300 °C in hydrogen, purged in helium, and then cooled to 50 °C prior to the reaction.

**Figure 7:**
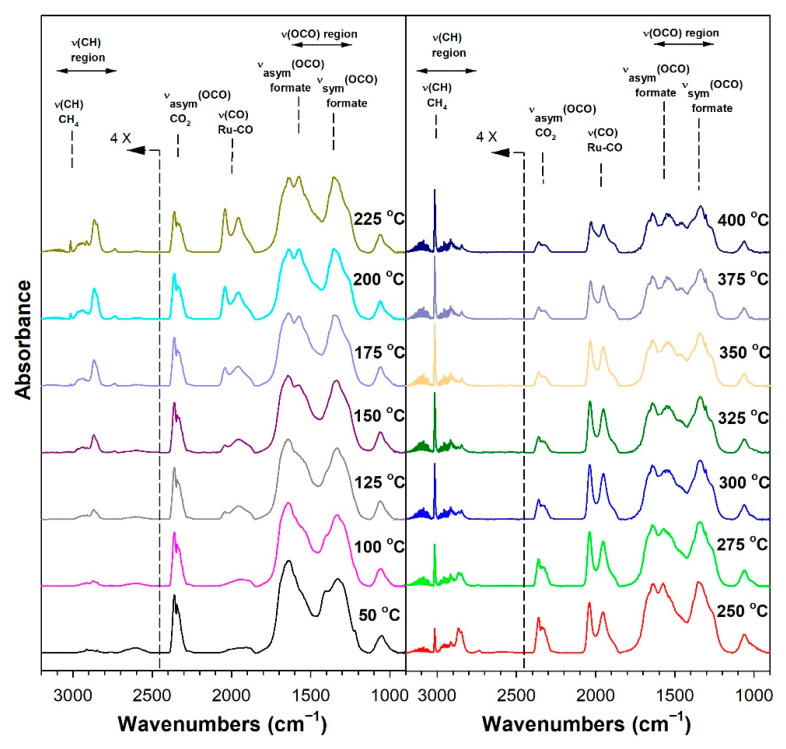
DRIFTS of CO_2_ hydrogenation with 4%CO_2_ + 12%H_2_ (balance He) over 0.5%Na-1%Ru/m-zirconia activated at 300 °C in hydrogen, purged in He, and then cooled to 50 °C prior to reaction.

**Figure 8:**
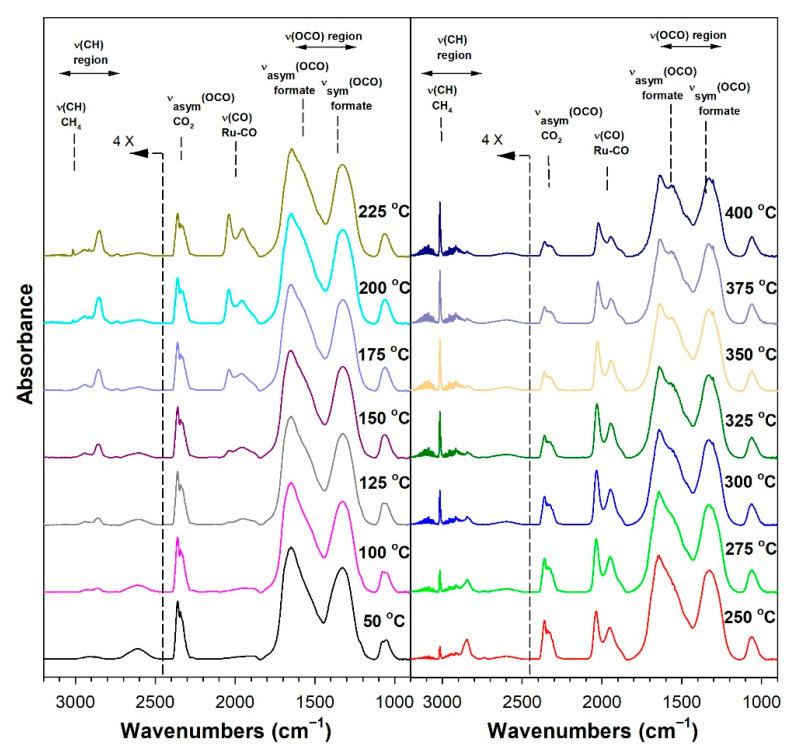
DRIFTS of CO_2_ hydrogenation with 4%CO_2_ + 12%H_2_ (balance He) over 1%Na-1%Ru/m-zirconia activated at 300 °C in hydrogen, purged in He, and then cooled to 50 °C prior to reaction.

**Figure 9:**
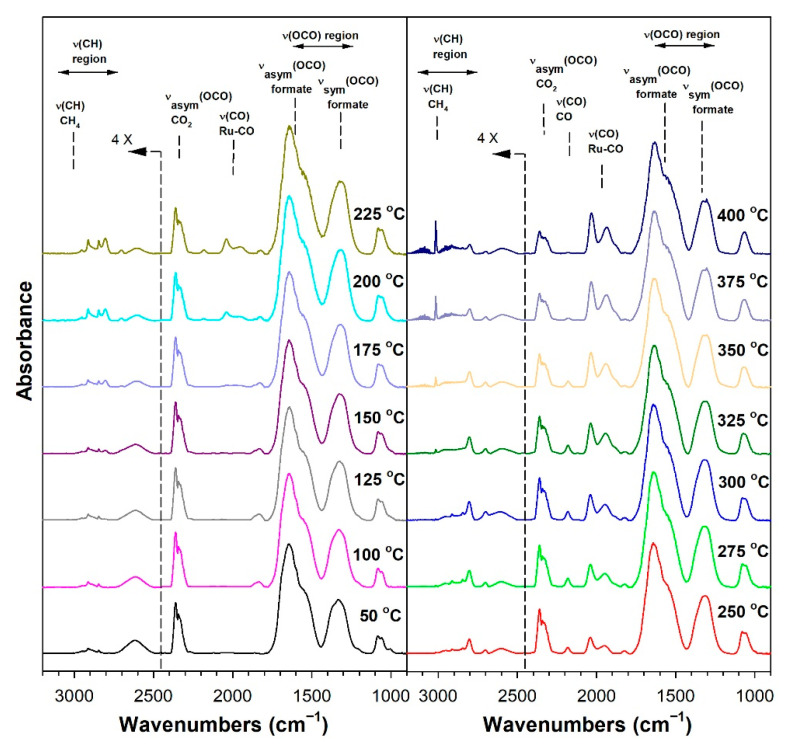
DRIFTS of CO_2_ hydrogenation with 4%CO_2_ + 12%H_2_ (balance He) over 1.8%Na-1%Ru/m-zirconia activated at 300 °C in hydrogen, purged in He, and then cooled to 50 °C prior to reaction.

**Figure 10:**
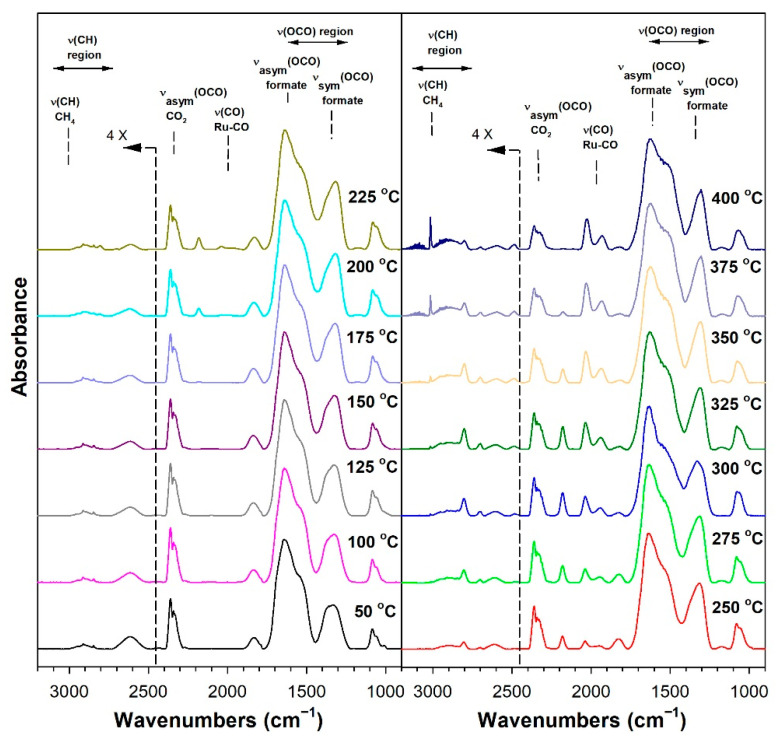
DRIFTS of CO_2_ hydrogenation with 4%CO_2_ + 12%H_2_ (balance He) over 2.5%Na-1%Ru/m-zirconia activated at 300 °C in hydrogen, purged in He, and then cooled to 50 °C prior to reaction.

**Figure 11:**
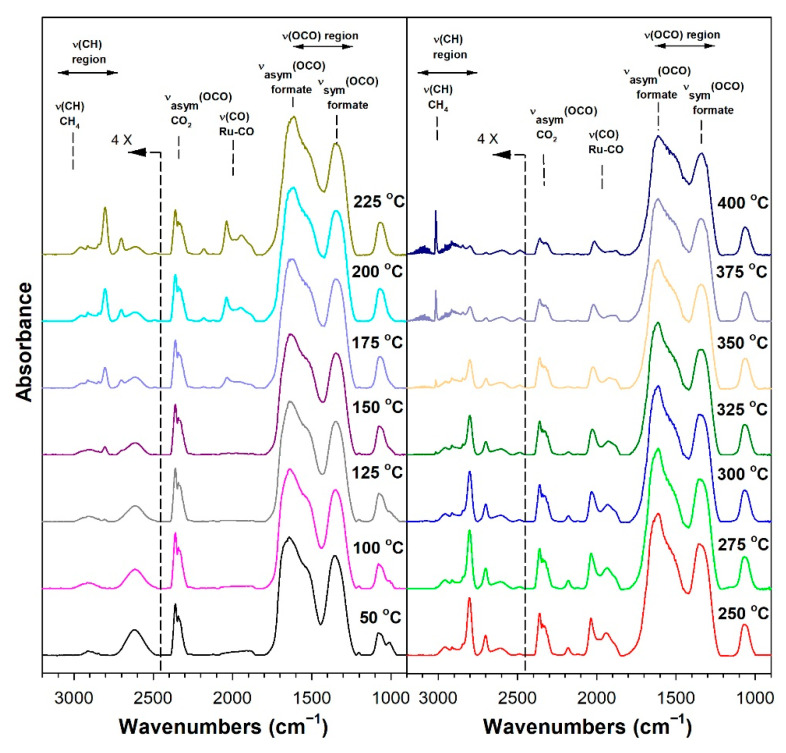
DRIFTS of CO_2_ hydrogenation with 4%CO_2_ + 16%H_2_ (balance He) over 2.5%Na-1%Ru/m-zirconia activated at 300 °C in hydrogen, purged in He, and then cooled to 50 °C prior to reaction.

**Figure 12:**
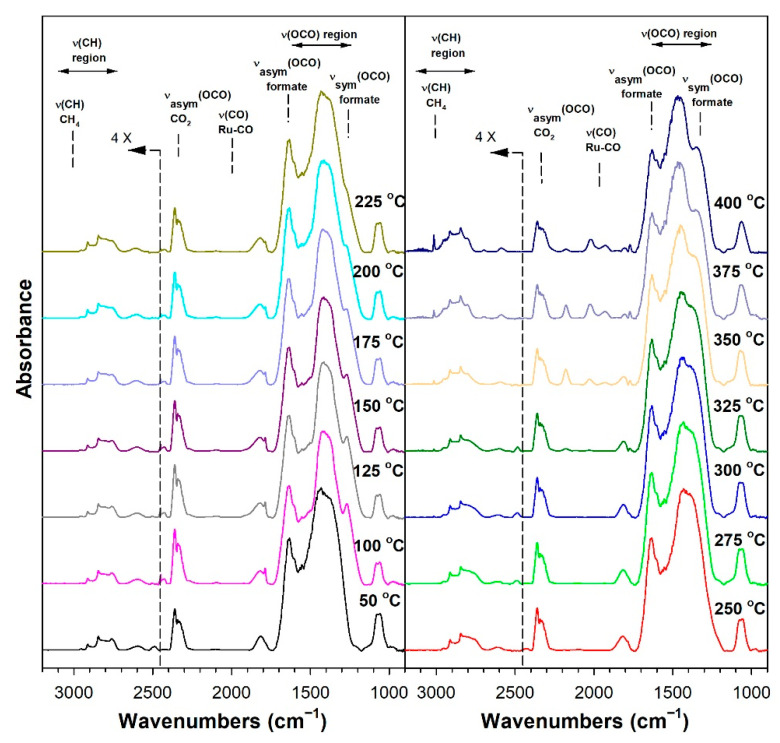
DRIFTS of CO_2_ hydrogenation with 4%CO_2_ + 12%H_2_ (balance He) over 5%Na-1%Ru/m-zirconia activated at 300 °C in hydrogen, purged in He, and then cooled to 50 °C prior to reaction.

Before conducting CO_2_ hydrogenation experiments using in-situ DRIFTS, control experiments of CO_2_ in He were performed (Figures S10 and S11 for 1%Ru/m-zirconia and 2.5%Na-1%Ru/m-zirconia, respectively). The ν(OCO) infrared bands of surface carbonates and/or bicarbonate species were observed over the entire temperature range. CO_2_ hydrogenation experiments were then carried for the purpose of characterizing the surface species formed and the temperature and the degree of methanation. The infrared spectra recorded during CO_2_ hydrogenation are shown in Figure 5, Figure 6, Figure 7, Figure 8, Figure 9, Figure 10, Figure 11 and Figure 12 for all catalysts. The Na doping effect can be observed by comparing Figure 5, Figure 7, Figure 8, Figure 9, Figure 10 and Figure 12, which were conducted at an H_2_:CO_2_ ratio equal to 3:1. The effect of the H_2_:CO_2_ ratio (i.e., 3:1 versus 4:1) can be examined by comparing Figure 5 and Figure 6 for undoped 1%Ru/m-zirconia and Figure 11 and Figure 12 for 2.5%Na-1%Ru/m-zirconia. The wavenumber assignments of the bands for the various species detected on the catalysts are provided in Table 4. The DRIFTS spectra revealed that carbonates and/or bicarbonate species were formed at 50 °C. With an increase in temperature, formate species were formed from the hydrogenation of surface carbonates, presumably at the Ru-support junction. The intensities of these bands typically reached close to the maximum prior to the detection of gas-phase CO and CH_4_, as observed by the ν(CO) of CO and the ν(CH) band of CH_4_.

**Table 2:** Results of fittings the EXAFS data * recorded at the Ru K-edge for the catalysts after activation in H_2_ for 2 h and cooling. Ranges: Δk = 3–10 Å^−1^; ΔR = 1.5–3.0 Å. S_0_^2^ was set at 0.90.

Sample Description	N Ru-Ru Metal	R Ru-Ru (Å) Metal	e_0_ (eV)	σ^2^ (Å^2^)	R-Factor	Calc. # Atoms *	Calc. Diam. (nm) *
Ruthenium foil	**12**	2.681 (0.0068)	−0.920 (1.457)	0.00370 (0.000282)	0.0095	-	-
0%Na-1%Ru/m-zirconia	2.0 (0.81)	2.674 (0.0178)	1.442 (4.440)	0.00294 (0.00254)	0.016	4.7 **	0.31
0.5%Na-1%Ru/m-zirconia	3.7 (0.47)	2.665 (0.0053)	0.592 (0.924)	0.00334 (0.000938)	0.0074	8.6	0.56
1%Na-1%Ru/m-zirconia	3.4 (0.42)	2.669 (0.0050)	0.133 (0.915)	0.00245 (0.000891)	0.0063	7.9	0.52
1.8%Na-1%Ru/m-zirconia	3.1 (0.64)	2.666 (0.0082)	1.484 (1.536)	0.00160 (0.00147))	0.017	7.2	0.47
2.5%Na-1%Ru/m-zirconia	3.7 (0.72)	2.671 (0.0084)	2.154 (1.372)	0.00437 (0.00147)	0.019	8.7	0.57
5%Na-1%Ru/m-zirconia	4.5 (0.32)	2.669 (0.0030)	−0.673 (0.523)	0.00354 (0.000529)	0.0023	10.6	0.69

* Estimated by comparison with Marinkovic et al. [51] using a near spherical morphology. ** Logically, ~3, as the extrapolation is more accurate at higher N values.

**Table 3:** Peak area % of Gaussian peaks, with peak maxima within various temperature ranges, used for curve fitting the CO_2_ TPD results.

Catalyst	% T < 250 °C	% 250 °C < T < 400 °C	% T > 400 °C
1%Ru/zirconia	65	25	10
0.5%Na-1%Ru/m-zirconia	50	25	25
1%Na-1%Ru/m-zirconia	28	24	48
1.8%Na-1%Ru/m-zirconia	20	18	62
2.5%Na-1%Ru/m-zirconia	16	23	61
5%Na-1%Ru/m-zirconia	10	21	69

**Table 4:** Infrared band positions observed during CO_2_ hydrogenation (H_2_:CO_2_ = 3:1) obtained using the work of Binet et al. [52] as a reference. Catalysts were activated in hydrogen at 300 °C. Formate band positions were obtained at temperatures 25 °C below those at which the CH_4_ ν(CH) band was detected: undoped, 125 °C; 0.5 wt.% sodium, 150 °C; 1 wt.% sodium, 150 °C; 1.8 wt.% sodium, 225 °C; 2.5 wt.% sodium, 250 °C; and 5 wt.% sodium, 275 °C. The Ru carbonyl ν(CO) band positions were obtained at 250 °C. Bold text represents major bands. Shoulder and weak band features are shown in parentheses.

Catalyst	Band Position (cm^−1^)				
	ν(C-H) Formate	ν(CO) Ru-CO	ν_asym_ (OCO) Formate	ν_sym_ (OCO) Formate	Δ(OCO)
1%Ru/m-ZrO_2_	**2872**, (2848)	**2042**, (1970), **1955**, (1900)	1631, 1609, **1567**, **1557**	(1417, 1386) **1358**, **1335**	199, 232
w/0.5%Na	**2869**, (2848)	**2040**, (1965), **1954**, (1900)	**1643**, **1576**, (1539)	(1384), **1337**, (1266)	239, 306
w/1%Na	**2859**, (2806)	**2038**, **1952**, (1900)	**1652**, (1604–1450)	**1327**	325
w/1.8%Na	**2846**, **2804**	**2039**, **1950**, 1825	**1652** (1580–1450)	**1316**	336
w/2.5%Na	2846, **2804**	**2038**, **1950**, **1827**	**1642**	(1365), **1315**	327
w/5%Na	**2846**, **2834–2700**	**1818**	**1638**	**1435–1230**	306

**Figure 13:**
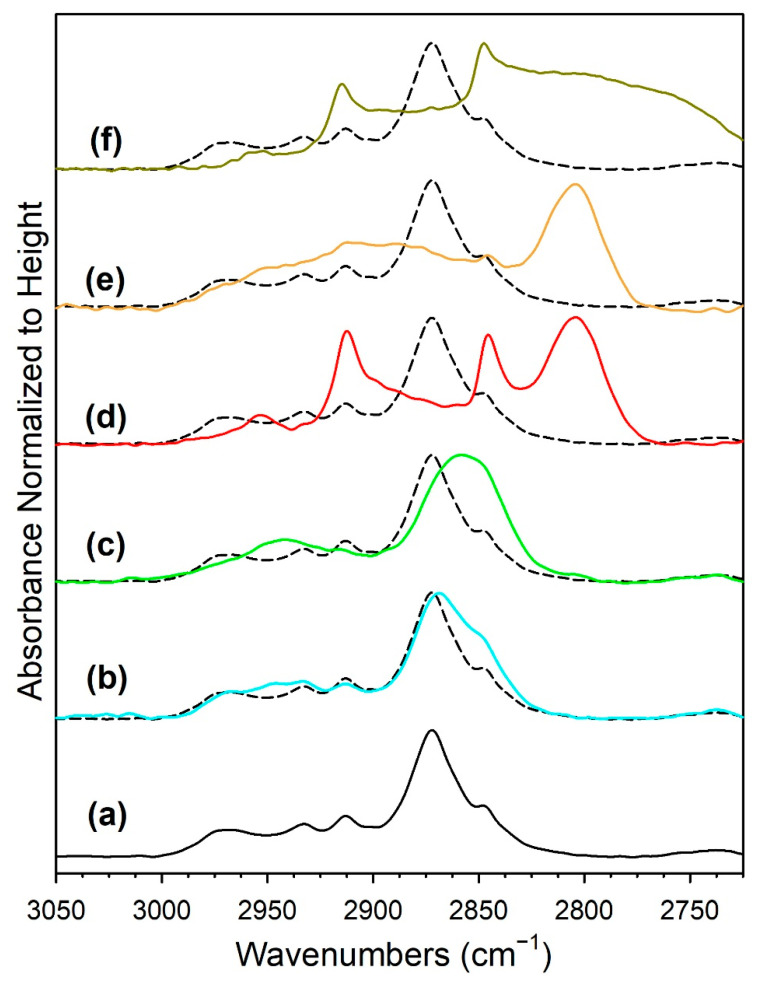
DRIFTS of the formate ν(CH) band, with the intensity normalized to the height, for CO_2_ hydrogenation with 4%CO_2_ + 12%H_2_ (balance He) over catalysts activated at 300 °C in H_2_, including (**a**, black) unpromoted 1%Ru/m-zirconia, and the promoted catalysts with Na loadings of (**b**, cyan) 0.5 wt.%, (**c**, green) 1%, (**d**, red) 1.8 wt.%, (**e**, orange) 2.5 wt.%, and (**f**, dark yellow) 5 wt.%. Spectra were recorded at temperatures 25 °C below those at which the n(CH) signal of CH_4_ was detected, including (**a**) 125 °C, (**b**) 150 °C (**c**) 150 °C, (**d**) 225 °C, (**e**) 250 °C, and (**f**) 275 °C. Dashed line is the unpromoted sample for reference.

Figure 13 shows the formate ν(CH) band with the intensity normalized to the height for the purpose of comparing positions. Table 4 shows the band positions. The results are in good agreement with the observations of our low pressure investigation [19]; however, in that study, the H_2_/CO_2_ ratio was 15:1, whereas in the current study the same H_2_/CO_2_ ratio as that used in fixed-bed reaction tests was employed. As observed in forward WGS experiments, the formate ν(CH) band position in RWGS was located at lower wavenumbers when the Na doping reached 1.8%Na (which is close to the optimal doping level for promoting the WGS reaction). From a loading of 1.8%Na to 2.5%Na, the band position changed very little (Δ < 1 cm^−1^) and then broadened at 5%Na. Table 4 also provides the formate band positions for the asymmetric and symmetric ν(OCO) modes. The difference in the band positions of the asymmetric and symmetric ν(OCO) bands provides information about the strength of bonding with the catalyst surface. With an increase in Na loading, there was an increase in the strength of this interaction, as the differences exhibited an increasing trend, as follows (note that, in some cases, multiple formate species were detected, and we report the primary bands observed): undoped, Δ = 199 and 232 cm^−1^; 0.5 wt.% Na, Δ = 239 and 306 cm^−1^; 1 wt.% Na, Δ = 325 cm^−1^; and 1.8 wt.% Na, Δ = 336 cm^−1^. Above 1.8 wt.% Na, the difference decreased somewhat: 2.5 wt.% Na, Δ = 327 cm^−1^ and 5 wt.% Na, Δ = 306 cm^−1^. These results align well with the trend observed with CO_2_ TPD, where the higher basicity of Na-doped catalysts led to shifts in the CO_2_ TPD profiles to higher temperatures. However, while that conclusion was likely affected by the inhibiting effect of Na on Ru-catalyzed CO_2_ removal, the splitting of the formate ν(OCO) bands was not impacted by the effect of Ru present in CO_2_ TPD, and as such, this may be a better indication of higher basicity with the addition of Na. Thus, as shown in our prior investigation at a low pressure [19], the DRIFTS and CO_2_ TPD results provide strong evidence that the catalyst basicity is increased with the addition of Na. With the addition of Na, there is an increase in the strength of the interaction between the O–C–O functional group and the catalyst surface. This is proposed to be the cause of C–H bond weakening, as strongly suggested by the formate ν(CH) band shift to lower wavenumbers. To reiterate, in the absence of other effects, formate C–H bond weakening should promote C–H bond scission in forward WGS (i.e., it is the proposed rate limiting step in forward WGS), accelerating the catalytic cycle. For CO_2_ hydrogenation via RWGS, according to the Principle of Microscopic Reversibility, Na doping should therefore (once again, in the absence of other effects) tend to promote C–H bond formation when producing the formate intermediate and, as a result, promote the formation of CO.

In in situ DRIFTS studies, the general mechanistic trend is that carbonate species are formed at low temperature; with an increasing temperature, carbonate is hydrogenated to formate intermediates, which decompose to CO with the aid of Ru. Once CO is formed, Na plays an important role in determining whether the CO is subsequently hydrogenated to CH_4_ by a secondary reaction, or whether it desorbs. The attenuation of methanation may also lead to a greater probability of chain growth at higher pressures.

**Figure 14:**
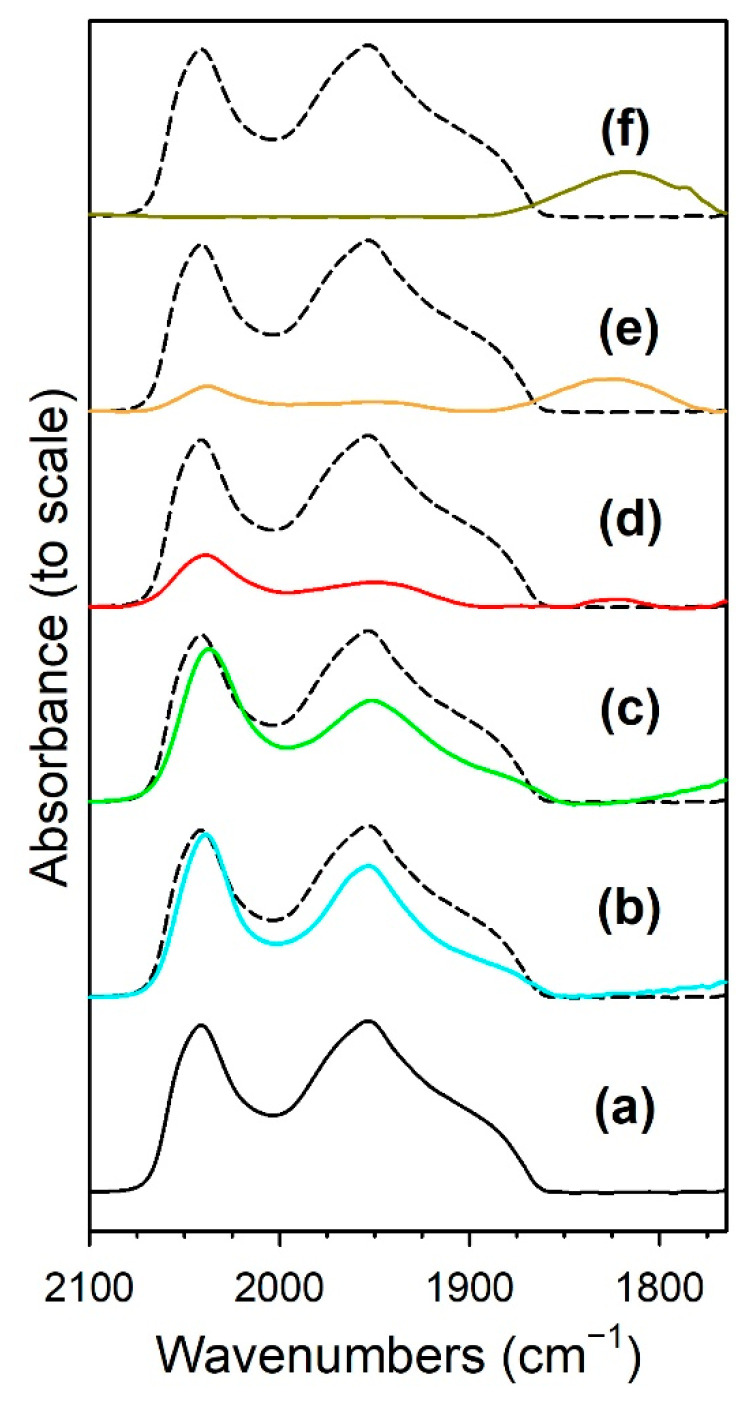
DRIFTS of Ru carbonyl ν(CO) bands for CO_2_ hydrogenation with 4%CO_2_ + 12%H_2_ (balance He) using catalysts activated at 300 °C in H_2_, including (**a**, black) unpromoted 1%Ru/m-zirconia, as well as promoted catalysts with Na loadings of (**b**, cyan) 0.5 wt.%, (**c**, green) 1 wt.%, (**d**, red) 1.8 wt.%, (**e**, orange) 2.5 wt.%, and (**f**, dark yellow) 5 wt.%. Spectra were recorded at 250 °C. Dashed line is the unpromoted sample for reference.

Figure 14 shows that, as in the case of the forward water–gas shift reaction described earlier, the Ru carbonyl intensity is attenuated by the addition of Na, as follows: undoped, 100%; 0.5 wt.% sodium, 75%; 1 wt.% sodium, 69%; 1.8 wt.% sodium, 19%; 2.5 wt.% sodium, 17%; 5 wt.% sodium, 14%. As such, methanation becomes suppressed by the disruption of ensembles of Ru surface atoms by Na. By examining the initial temperature of CH_4_ formation (Table 5) through the ν(CH) band of CH_4_ at 3010–3020 cm^−1^, the following trend was observed: undoped, 150 °C; 0.5 wt.% sodium, 175 °C; 1 wt.% sodium, 175 °C; 1.8 wt.% sodium, 250 °C 2.5 wt.% sodium, 275 °C; and 5 wt.% sodium, 300 °C. Thus, the addition of sodium inhibited methanation remarkably, shifting its initial formation temperature by Δ = +150 °C.

There was also a shift in the ruthenium carbonyl band to lower wavenumbers. From the geometric perspective, the addition of Na may diminish the dipole coupling of CO, resulting in a shift of the ruthenium carbonyl bands to lower wavenumbers. In that case, the role of Na may be to decrease the surface concentration and mobility of hydrogen adsorbed on the surfaces of Ru metal nanoparticles, as proposed by Komaya et al. for FTS over Na-doped Ru/TiO_2_ catalysts [36] and Jacobs et al. for Ag and Au promoted Co/Al_2_O_3_ FTS catalysts [39]. From an electronic point of view, alkali promoters have been proposed to donate electron density to the catalyst surface and, in turn, facilitate the backdonation of electron density from the catalyst surface to the 2π* antibonding molecular orbital of CO, weakening the CO bond and shifting the carbonyl bands to lower wavenumbers. Görling et al. [53] determined that about half of the shift to lower wavenumbers is due to electronic backdonation [54], while the additional shift is primarily due to electrostatic interactions between CO and the surface dipole layer modified by alkali. With the related Na-doped Pt/m-ZrO_2_ system [55], we did not obtain evidence for an electron transfer effect in applying the XANES difference procedure at the the L_III_ minus L_II_ edges of Pt. Although we could not apply that method to the Ru system, we did not observe any change in the XANES line shape profile or observe a shift in the K-edge energy that would confirm such an electronic effect. Nevertheless, an electronic effect cannot be ruled out.

Thus, Na likely plays two important roles in controlling the selectivity. Weakening the carbon–hydrogen bond of formate tends to promote the RWGS cycle, while attenuating the Ru metal activity by the addition of Na inhibits methanation. Based on the loading of Na, the relative rates of CO and CH_4_ formation can therefore be controlled to a significant degree. The inhibition of methanation also means that the probability of C–C coupling may be improved when CO_2_ hydrogenation is conducted at higher pressures.

#### 3.1.7. Temperature-Programmed Surface Reaction with Mass Spectrometry

**Figure 15:**
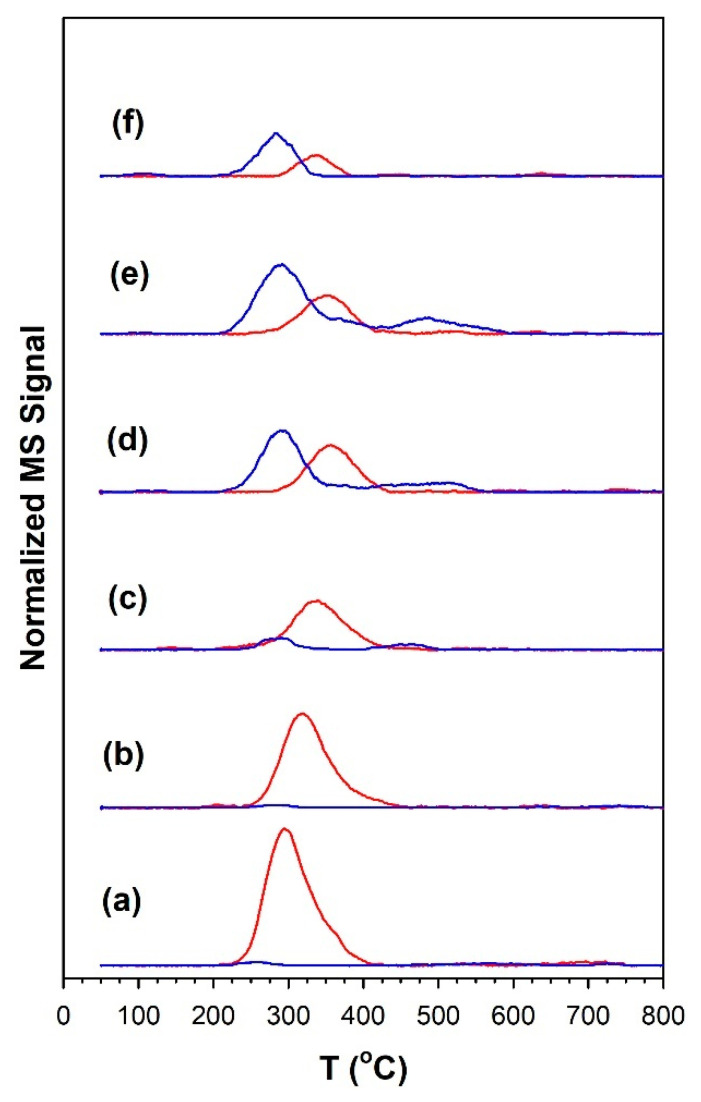
TP-rxn/MS of adsorbed 4%CO_2_/12%H_2_ (balance He), including the MS signals of (blue) CO and (red) CH_4_, for (**a**) unpromoted 1%Ru/m-ZrO_2_ and the same catalyst doped with (**b**) 0.5%Na, (**c**) 1%Na, (**d**) 1.8%Na, (**e**) 2.5%Na, and (**f**) 5%Na.

The TP-rxn/MS results for the CO_2_ hydrogenation of preadsorbed 4%CO_2_/12%H_2_ are reported in Figure 15 and Table 6. For the undoped 1%Ru/m-zirconia catalyst and the same catalyst promoted with up to 1%Na, the profile was dominated by CH_4_ (red curves), while for catalysts with 1.8%Na to 5%Na, the profile switched and was dominated by CO (blue curves). As shown in Table 6, in all cases, the maximum temperature for CO evolution was below the maximum temperature for CH_4_ evolution, with the difference being in the range of 37–64 °C. Combined, these two results strongly suggest that the mechanism involves, to a significant degree, a stepwise mechanism involving the primary formation of CO (or adsorbed CO) followed by the interception of CO for secondary hydrogenation (e.g., at low P, to CH_4_). Na thus acts to profoundly facilitate CO formation (i.e., RWGS) and inhibit the secondary hydrogenation reaction (e.g., methanation).

#### 3.1.8. Catalytic Testing

**Figure 16:**
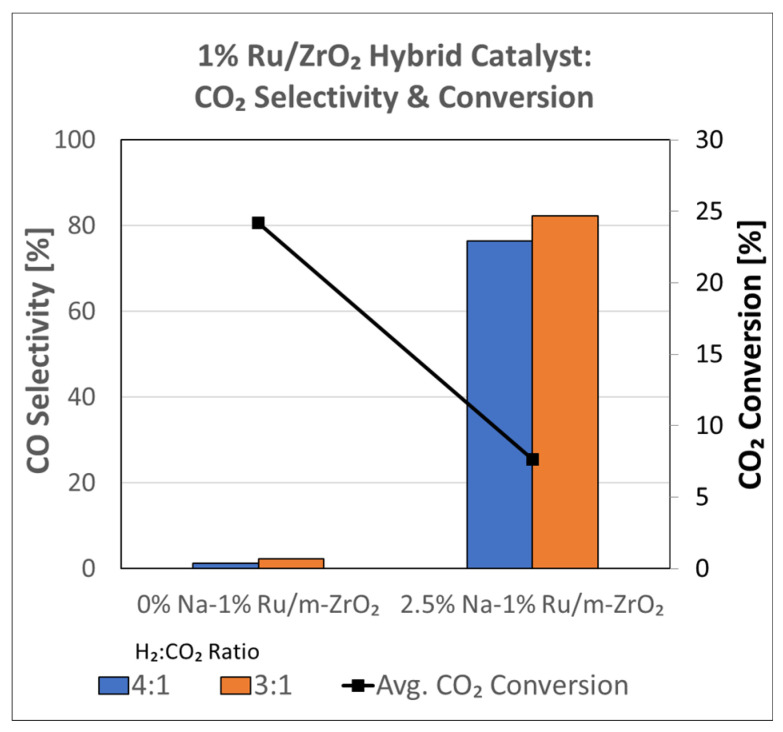
Effect of the H_2_:CO_2_ ratio on reaction testing at 1 bar and 300 °C at a space velocity of 60,000 mL/g_cat_/h using a fixed-bed reactor with 1%Ru/m-zirconia versus 2.5%Na-1%Ru/m-zirconia catalysts, including ratios of (blue) 4:1 and (orange) 3:1.

Prior to carrying out reaction testing at 20 bar, the influence of the H_2_:CO_2_ ratio on the methanation selectivity was investigated for the undoped versus 2.5%Na-promoted catalysts. In our prior investigation at a low pressure, the H_2_:CO_2_ ratio was 4:1; however, we were interested in determining whether operating at a H_2_:CO_2_ ratio equal to 3:1 (stoichiometric for RWGS/Fischer–Tropsch) might diminish the methanation selectivity at a low pressure. Comparing Figure 5 (H_2_:CO_2_ = 3:1) and 6 (H_2_:CO_2_ = 4:1) for unpromoted 1%Ru/m-ZrO_2_, the primary difference is not the temperature at which methane forms, but rather, that the methane bands are stronger in intensity at an H_2_:CO_2_ ratio of 4:1. The same is true when comparing Figure 10 and Figure 11 for the 2.5%Na-promoted catalyst. Testing the undoped and 2.5%Na-promoted catalyst in the fixed-bed reactor at a low pressure (Figure 16), the CO selectivity was shown to be slightly improved (~1%) for the unpromoted catalyst and more significantly improved (~6%) for the 2.5%Na-promoted catalyst after switching to the lower H_2_/CO_2_ ratio of 3:1 from 4:1. As a result, the H_2_:CO_2_ ratio of 3:1 was used (stoichiometric for RWGS/Fischer-Tropsch) to carry out tests at the higher pressure of 20 bar.

**Figure 17:**
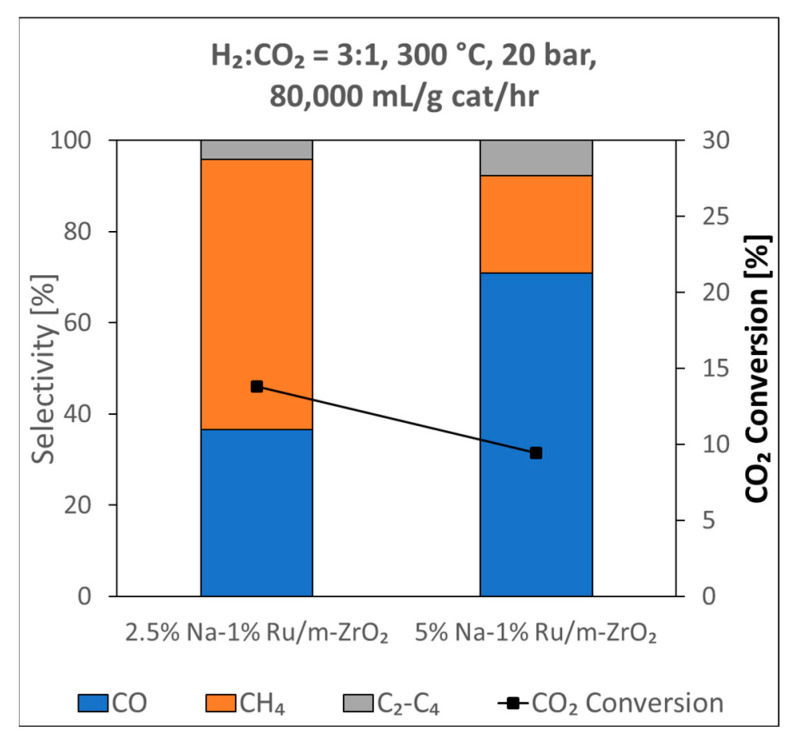
Effect of the Na-doping level on reaction testing in a fixed bed reactor at 20 bar, 300 °C, and a H_2_:CO_2_ ratio of 3:1 for 2.5%Na-1%Ru/m-ZrO_2_ and 5%Na-1%Ru/m-ZrO_2_ catalysts.

Hybrid Na-promoted 1%Ru/m-ZrO_2_ catalysts comprised of a RWGS function and an FTS function were tested at an elevated pressure of 20 bar, a temperature of 300 °C, a space velocity equal to 80,000 mL/g_cat_∙h, and an H_2_/CO_2_ ratio 3:1. As shown in Figure 17, increasing the Na content from 2.5 wt.% sodium to 5 wt.% sodium slightly decreased the CO_2_ conversion from ~14% to ~10%. However, the selectivity for desired products was greatly improved. Undesired methanation decreased, with CH_4_ selectivity dropping from ~60% to ~21%, while desired CO selectivity almost doubled from ~36% to ~71%, and desired chain growth product selectivity (C_2_–C_4_ selectivity) doubled from ~4% to ~8%.

## 4. Conclusions

By promoting 1%Ru/m-zirconia with sodium and adjusting the H_2_:CO_2_ ratio from 4:1 to 3:1, the selectivity of the CO_2_ hydrogenation reaction was turned away from methanation to favor the RWGS product, CO. At an elevated pressure of 20 bar, in addition to improvements in the CO selectivity, the selectivity to chain growth products was significantly improved as well. For example, at 20 bar, by increasing the Na content from 2.5 wt.% to 5 wt.%, the CH_4_ selectivity was decreased by more than half, from 60% to 21%, while the CO selectivity increased from 36% to 71% and that of light hydrocarbons (C_2_–C_4_) doubled from 4% to 8%. CO_2_ TPD and an analysis of the splitting of the formate ν(OCO) bands during CO_2_ hydrogenation showed that the addition of Na to the catalyst (as well as increasing the Na doping level) increased the basicity of the catalyst. This increase in basicity from Na doping resulted in a weakening of the formate intermediate C–H bond, as confirmed by an observed shift in the ν(CH) band of formate to lower wavenumbers. While this facilitates formate C–H bond scission during forward WGS, boosting CO conversion, during reverse WGS, this effect promotes the formation of carbon–hydrogen bonds when producing the formate intermediate, and in turn, facilitates the formation of CO at the Ru/m-zirconia junction. At the same time, increasing the amount of Na dopant attenuates the hydrogenation activity of Ru^0^ at on-top sites, and systematic decreases in Ru carbonyl intensity were observed in infrared spectroscopy during CO_2_ hydrogenation. Moreover, the site capacity, as measured by H_2_ chemisorption, was found to be significantly lower for the 2.5 wt.% and 5 wt.% Na-promoted catalysts, despite all catalysts having small particle sizes, as measured by EXAFS, that should, in the absence of coverage by Na, otherwise have led to high dispersion. Because these Ru^0^ on-top sites are responsible for intercepting CO from the Ru/m-zirconia junction and subsequently converting the CO formed to methane, the addition of sodium tends to suppress the methanation reaction. An added benefit is that suppressing the excessive hydrogenation activity of Ru^0^ increases the probability of C–C chain growth, thereby boosting the selectivity of C_2_–C_4_ products. Thus, if the desired product is synthetic natural gas, the addition of Na should be avoided. On the other hand, if CO is desired for making syngas (to be used in downstream Fischer–Tropsch synthesis or methanol-to-gasoline processes), then adding sodium to the catalyst is beneficial, and some chain growth products can be made during the RWGS section as well. The first primary role of Na is to enhance the catalyst basicity, and this has a direct effect on the weakening of the formate carbon–hydrogen bond by increasing the strength of the interaction between the catalyst surface and the O–C–O functional group of formate. As a result, Na doping facilitates the formation of surface formate intermediates which, in turn, promotes CO production via RWGS. The second primary role of Na is to suppress the excessive hydrogenation activity of Ru^0^ metal sites, either allowing CO to escape further hydrogenation or increasing the probability of C-C chain growth, promoting the formation of C_2_–C_4_ products.

## Figures and Tables

**Table 1:** Main TPR peaks in °C.

Catalyst	Main Peaks, T (°C)
1%Ru/m-zirconia	187
Same with: 0.5%Na	218
1%Na	222, 234
1.8%Na	234, 272
2.5%Na	235, 327
5%Na	238, 349, 390, 402

**Table 5:** Temperature of initial CH_4_ formation under CO_2_ hydrogenation (H_2_:CO_2_ = 3:1) using DRIFTS.

Catalyst	T (°C) at Which the ν(CH) of CH_4_ Was Detected
1%Ru/m-zirconia	150
same with 0.5 wt.% Na	175
1 wt.% Na	175
1.8 wt.% Na	250
2.5 wt.% Na	275
5 wt.% Na	300

**Table 6:** Results of TP-rxn/MS (H_2_:CO_2_ = 3:1) compiled from Figure 15.

Catalyst	T_max_ (°C) at Which CO Was Detected	T_max_ (°C) at Which CH_4_ Was Detected	CO/CH_4_ Signal Ratio
1%Ru/m-ZrO_2_	251	296	<0.05
w/0.5%Na	280	317	<0.05
w/1%Na	291	339	0.33
w/1.8%Na	291	355	1.5
w/2.5%Na	291	355	2.3
w/5%Na	283	339	2.4

## Data Availability

Not applicable.

## Supplementary Materials

The following supporting information can be downloaded at: https://www.mdpi.com/article/10.3390/nano13071155/s1, Table S1: BET surface area; Figure S1: Hydrogen chemisorption by TPD; Figure S2: H_2_-TPR/TPR-MS; Figure S3: Linear combination fitting of TPR-XANES; Figure S4: XANES overlays following H_2_-TPR; Figure S5: TPR-EXAFS Fourier transform magnitude spectra; Figure S6: DRIFTS of the formate ν(CH) band following CO adsorption; Figure S7: Forward formate decomposition in H_2_O; Figure S8: Effect of Na on attenuating the Ru carbonyl ν(CO) band intensity, Figure S9: Ru carbonyl decomposition in H_2_O; Figure S10: DRIFTS of 4%CO_2_ adsorption for unpromoted 1%Ru/m-zirconia; Figure S11: DRIFTS of 4%CO_2_ adsorption for 2.5%Na-1%Ru/m-zirconia.

## References

[1] Alvez L.M.N.C., Almeida M.P., Ayala M., Watson C.D., Jacobs G., Rabelo-Neto R.C., Noronha F.B., Mattos L.V. (2021). CO_2_ methanation over metal catalysts supported on ZrO_2_: Effect of the nature of the metallic phase on catalytic performance. Chem. Eng. Sci..

[2] Xie Y., Chen J., Wu X., Wen J., Zhao R., Li Z., Tian G., Zhang Q., Ning P., Hao J. (2022). Frustrated Lewis pairs boosting low-temperature CO_2_ methanation performance over Ni/CeO_2_ nanocatalysts. ACS Catal..

[3] Shafer W.D., Jacobs G., Graham U.M., Hamdeh H.H., Davis B.H. (2019). Increased CO_2_ hydrogenation to liquid products using promoted iron catalysts. J. Catal..

[4] Visconti C., Martinelli M., Falbo L., Fratalocchi L., Lietti L. (2016). CO_2_ hydrogenation to hydrocarbons over Co and Fe-based Fischer-Tropsch catalysts. Catal. Today.

[5] Atsbha T.A., Yoon T., Seongho P., Lee C.-J. (2021). A review on the catalytic conversion of CO_2_ using H_2_ for synthesis of CO, methanol, and hydrocarbons. J. CO2 Util..

[6] Santiago R.G., Coelho J.A., De Lucena S.M.P., Musse A.P.S., Portilho M.D.F., Rodriguez-Castellon E., De Azevedo D.C.S., Bastos-Neto M. (2022). Synthesis of MeOH and DME from CO_2_ hydrogenation over commercial and modified catalysts. Front. Chem..

[7] Panahi M., Karimi M., Skogestad S., Hillestad M., Svendsen H.F. (2010). Self-optimizing and control structure design for a CO_2_ capturing plant. Adv. Gas Proc..

[8] Fugiel A., Burchart-Korol D., Czaplicka-Kolarz K., Smolinski A. (2017). Environmental impact and damage categories caused by air pollution emissions from mining and quarrying sectors of European countries. J. Clean. Prod..

[9] Soto V., Ulloa C., Garcia X. (2021). A CFD design approach for industrial size tubular reactors for SNG production from biogas (CO_2_ Methanation). Energies.

[10] Wang T., Wang X., Hou C., Liu J. (2020). Quaternary functionalized mesoporous adsorbents for ultra-high kinetics of CO_2_ capture from air. Sci. Rep..

[11] Straatman P.J.T., van Sark W.J.H.M. (2021). Indirect air CO_2_ capture and refinement based on OTEC seawater outgassing. iScience.

[12] Li G., Li J., Dai Z., Akram M.W. (2022). Modelling and analysis of a novel hydrogen production approach by full spectrum solar energy. Energy Conv. Manag..

[13] Goekcek M. (2010). Hydrogen generation from small-scale wind-powered electrolysis system in different power matching modes. Int. J. Hydrogen Energy.

[14] Sahin S., Sahin H.M. (2021). Generation-IV reactors and nuclear hydrogen production. Int. J. Hydrogen Energy.

[15] Guarieiro L.L.N., dos Anjos J.P., da Silva L.A., Santos A.A.B., Calixto E.E.S., Pessoa F.L.P., de Almeida J.L.G., Filho M.A., Marinho F.S., da Rochac G.O. (2022). Technological perspectives and economic aspects of green hydrogen in the energetic transition: Challenges for chemistry. J. Braz. Chem. Soc..

[16] Pahija E., Panaritis C., Rutherford B., Couillard M., Patarachao B., Shadbahr J., Bensebaa F., Patience G.S., Boffito D.C. (2022). FeO_X_ nanoparticle doping on Cu/Al_2_O_3_ catalysts for the reverse water gas shift. J. CO2 Util..

[17] Nielsen A.S., Ostadi M., Austboe B., Hillestad M., del Alamo G., Burheim O. (2021). Enhancing the efficiency of power- and biomass-to-liquid fuel processes using fuel-assisted solid oxide electrolysis cells. Fuel.

[18] Kianfar E., Mazaheri H. (2020). Methanol to gasoline: A sustainable transport fuel. Adv. Chem. Res..

[19] Rabelo-Neto R.C., Almeida M.P., Silveira E.B., Ayala M., Watson C.D., Villarreal J., Cronauer D.C., Kropf A., Martinelli M., Noronha F.B. (2022). CO_2_ hydrogenation: Selectivity control of CO versus CH_4_ achieved using Na doping over Ru/m-ZrO_2_ at low pressure. Appl. Catal. B Environ..

[20] Gnanamani M., Hamdeh H.H., Jacobs G., Shafer W.D., Hopps S.D., Thomas G.A., Davis B.H. (2017). Hydrogenation of carbon dioxide over K-promoted FeCo bimetallic catalysts prepared from mixed metal oxalates. ChemCatChem.

[21] Numpilai T., Cheng C.K., Limtrakul J., Witoon T. (2021). Recent advances in light olefins production from catalytic hydrogenation of carbon dioxide. Proc. Saf. Environ. Protect..

[22] Pendyala V.R.R., Jacobs G., Graham U.M., Shafer W.D., Martinelli M., Kong L., Davis B.H. (2017). Fischer-Tropsch synthesis: Influence of acid treatment and preparation method on carbon nanotube supported ruthenium catalysts. Ind. Eng. Chem. Res..

[23] Porta A., Falbo L., Visconti C.G., Lietti L., Bassano C., Deiana P. (2020). Synthesis of Ru-based catalysts for CO_2_ methanation and experimental assessment of intraporous transport limitations. Catal. Today.

[24] Jacobs G., Davis B.H. (2010). Surface interfaces in low temperature water-gas shift: The metal-oxide synergy, the assistance of co-adsorbed water, and alkali doping. Int. J. Hydrogen Energy.

[25] Chenu E., Jacobs G., Crawford A.C., Keogh R.A., Patterson P.M., Sparks D.E., Davis B.H. (2005). Water-gas shift: An examination of Pt promoted MgO and tetragonal and monoclinic ZrO_2_ by in-situ DRIFTS. Appl. Catal. B Environ..

[26] Hagemeyer A., Carhart R.E., Yaccato K., Lesik A., Brooks C.J., Phillips C.B. (2010). Platinum-Alkali/Alkaline-Earth Catalyst Formulations for Hydrogen Generation. U.S. Patent.

[27] Shido T., Iwasawa Y. (1993). Reactant-promoted reaction mechanism for water-gas shift reaction of rhodium-doped ceria. J. Catal..

[28] Jacobs G., Graham U.M., Chenu E., Patterson P.M., Dozier A., Davis B.H. (2005). Low temperature water-gas shift: Impact of Pt promoter loading on the partial reduction of ceria, and consequences for catalyst design. J. Catal..

[29] Pigos J.M., Brooks C.J., Jacobs G., Davis B.H. (2007). Low temperature water-gas shift: Characterization of Pt-based ZrO_2_ catalyst promoted with Na discovered by combinatorial methods. Appl. Catal. A General.

[30] Pigos J.M., Brooks C.J., Jacobs G., Davis B.H. (2007). Low temperature water-gas shift: The effect of alkali doping on the C-H bond of formate over Pt/ZrO_2_ catalysts. Appl. Catal. A Gen..

[31] Martinelli M., Alhraki N., Castro J.D., Matamoros M.E., Jacobs G., Nanda S., Vo D.-V., Tri P.N. (2020). Effect of Na loading on Pt/ZrO_2_ catalysts for low temperature water-gas shift for the production and purification of hydrogen. New Dimensions in Production and Utilization of Hydrogen.

[32] Luukkanen S., Haukka M., Kallinen M., Pakkanen T.A. (2000). The low-temperature water-gas shift reaction catalyzed by sodium-carbonate-activated ruthenium mono (bipyridine)/SiO_2_ complexes. Catal. Lett..

[33] Basinska A., Domka F. (1997). The influence of alkali metals on the activity of supported ruthenium catalysts for the water-gas shift reaction. Catal. Lett..

[34] Pendyala V.R.R., Shafer W.D., Jacobs G., Graham U.M., Khalid S., Davis B.H. (2015). Fischer-Tropsch synthesis: Effect of reducing agent for aqueous-phase synthesis over Ru nanoparticle and supported Ru catalysts. Catal. Lett..

[35] Wang C., Zhao H., Wang H., Liu L., Xiao C., Ma D. (2012). The effects of ionic additives on the aqueous-phase Fischer–Tropsch synthesis with a ruthenium nanoparticle catalyst. Catal. Today.

[36] Komaya T., Bell A.T., Wengsieh Z., Gronsky R., Engelke F., King T.S., Pruski M. (1995). Effects of sodium on the structure and Fischer-Tropsch synthesis activity of Ru/TiO_2_. J. Catal..

[37] Williams F.J., Lambert R.M. (2000). A study of sodium promotion in Fischer–Tropsch synthesis: Electrochemical control of a ruthenium model catalyst. Catal. Lett..

[38] VanderWiel D.P., Pruski M., King T.S. (1999). A kinetic study on the adsorption and reaction of hydrogen over silica-supported ruthenium and silver–ruthenium catalysts during the hydrogenation of carbon monoxide. J. Catal..

[39] Jacobs G., Ribeiro M.C., Ma W., Ji Y., Khalid S., Sumodjo P.T.A., Davis B.H. (2009). Group 11 (Cu, Ag, Au) promotion of 15% Co/Al_2_O_3_ Fischer-Tropsch synthesis catalysts. Appl. Catal. A Gen..

[40] Datye A.K., Schwank J. (1985). Fischer-Tropsch synthesis on bimetallic ruthenium-gold catalysts. J. Catal..

[41] McCue A.J., Aponaviciute J., Wells R.P.K., Anderson J.A. (2013). Gold modified cobalt-based Fischer-Tropsch catalysts for conversion of synthesis gas to liquid fuels. Front. Chem. Sci. Eng..

[42] Jacoby M. (2001). X-ray absorption spectroscopy. Chem. Eng. News.

[43] Ressler T. (1998). WinXAS: A Program for X-ray Absorption Spectroscopy Data Analysis under MS-Windows. J. Synchrotron Radiat..

[44] Ravel B. (2001). ATOMS: Crystallography for the X-ray absorption spectroscopist. J. Synchrotron Rad..

[45] Newville M., Ravel B., Haskel D., Rehr J.J., Stern E.A., Yacoby Y. (1995). Analysis of multiple-scattering XAFS data using theoretical standards. Phys. B Condens. Matter.

[46] Fisher I.A., Bell A.T. (1998). In situ infrared study of methanol synthesis from H_2_/CO over Cu/SiO_2_ and Cu/ZrO_2_/SiO_2_. J. Catal..

[47] Kondo J., Sakata Y., Domen K., Maruya K., Onishi T. (1990). Infrared study of hydrogen adsorbed on ZrO_2_. J. Chem. Soc. Faraday Trans..

[48] Laachir A., Perrichon V., Badri A., Lamotte J., Catherine E., Lavalley J.C., El Fallah J., Hilaire L., Le Normand F., Quemere E. (1991). Reduction of CeO_2_ by Hydrogen. Magnetic Susceptibility and Fourier-Transform IR, UV and X-ray Photoelectron Spectroscopy Measurements. J. Chem. Soc. Faraday Trans..

[49] McHugh B.J., Larsen G., Haller G.L. (1990). Characterization of platinum particle interaction with L-zeolite by X-ray absorption spectroscopy: Binding energy shifts, X-ray absorption near-edge structure, and extended X-ray absorption fine structure. J. Phys. Chem..

[50] Bunluesin T., Gorte R.J., Graham G.W. (1998). Studies of the water-gas-shift reaction on ceria-supported Pt, Pd, and Rh: Implications for oxygen-storage properties. Appl. Catal. B Environ..

[51] Marinkovic N.S., Sasaki K., Adzic R. (2016). Nanoparticle size evaluation of catalysts by EXAFS: Advantages and limitations. Zast. Mater..

[52] Binet C., Daturi M., Lavalley J.C. (1999). IR study of polycrystalline ceria properties in oxidized and reduced states. Catal. Today.

[53] Görling A., Ackermann L., Lauber J., Knappe P., Rösch N. (1993). On the coadsorption of CO and alkali atoms at transition metal surfaces: A LCGTO-LDF cluster study. Surf. Sci..

[54] Blyholder G. (1964). Molecular orbital view of chemisorbed carbon monoxide. J. Phys. Chem..

[55] Martinelli M., Castro J.D., Alhraki N., Matamoros M.E., Kropf A.J., Cronauer D.C., Jacobs G. (2021). Effect of sodium loading on Pt/ZrO_2_ during ethanol steam reforming. Appl. Catal. A Gen..

