# Supplementary material for: CO2 Hydrogenation: Na Doping Promotes CO and Hydrocarbon Formation over Ru/m-ZrO2 at Elevated Pressures in Gas Phase Media
Source: Nanomaterials (Basel). 2023 Mar 24;13(7):1155. doi: 10.3390/nano13071155 (PMC10096969; doi:10.3390/nano13071155)
Supplement: Supplementary file 1 [file nanomaterials-13-01155-s001.zip › nanomaterials-2280858-supplementary.pdf]

Table S1: BET surface area, specific volume, and average pore diameter.

Sample ID	Measured A_s (BET) (m^2/g)	Expected A_s (BET) (m^2/g)	V_P (BJH Des) (cm^3/g)	D_P (BJH Des) (nm)
m-ZrO_2	95.4	95.4	0.289	9.5
1%Ru/m-ZrO_2	90.4	94.1	0.267	9.3
0.5%Na-1%Ru/m-ZrO_2	87.5	91.2 - 93.0	0.255	9.2
1.0%Na-1%Ru/m-ZrO_2	81.6	88.2 - 91.9	0.252	9.1
1.8%Na-1%Ru/m-ZrO_2	73.3	83.5 - 90.2	0.231	9.2
2.5%Na-1%Ru/m-ZrO_2	67.0	79.3 - 88.6	0.211	9.3
5.0%Na-1%Ru/m-ZrO_2	44.7	64.5 - 83.1	0.148	10.1

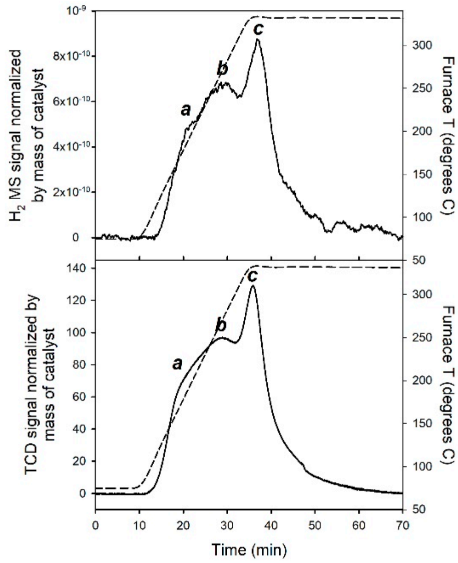

(a)

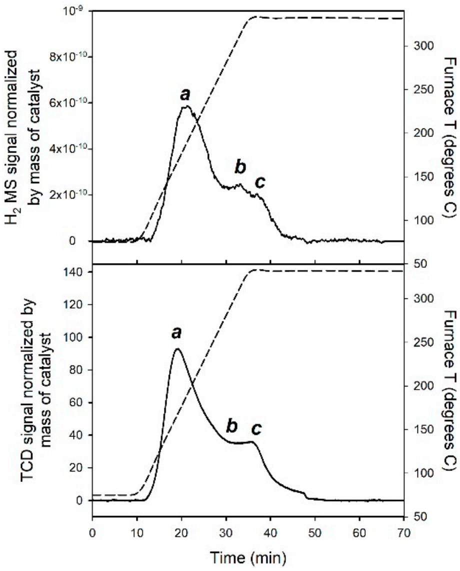

(b)

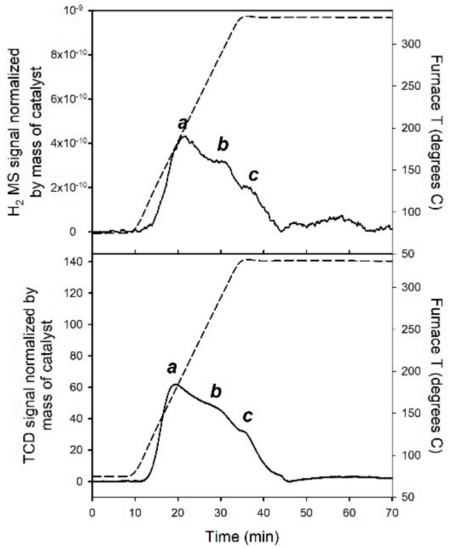

(c)

Figure S1: Results of hydrogen TPD for (a) 1%Ru/m-zirconia, (b) 2.5%Na-1%Ru/m-zirconia, and (c) 5%Na-1%Ru/m-zirconia. Peaks ***a***, ***b***, and ***c*** represent active sites of increasing chemisorption strength.

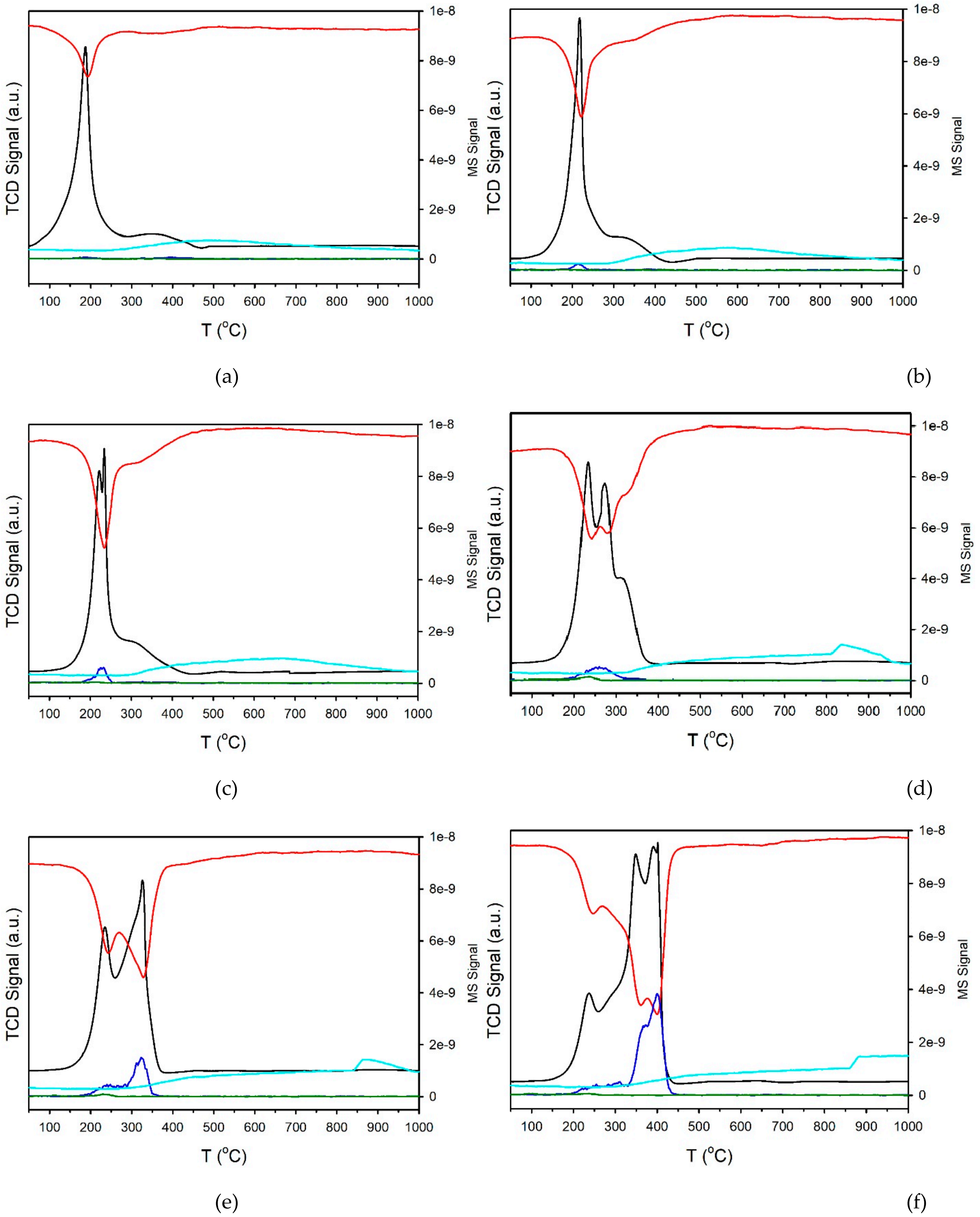

Figure S2: Hydrogen-TPR/ TPR-MS for (a) unpromoted 1%Ru/m-zirconia and the promoted catalyst with sodium loadings of (b) 0.5%, (c) 1%, (d) 1.8%, (e) 2.5%, and (f) 5%, including (black) TCD signal, and MS data for (red) hydrogen, (cyan) water, (blue) carbon monoxide, and (green) carbon dioxide.

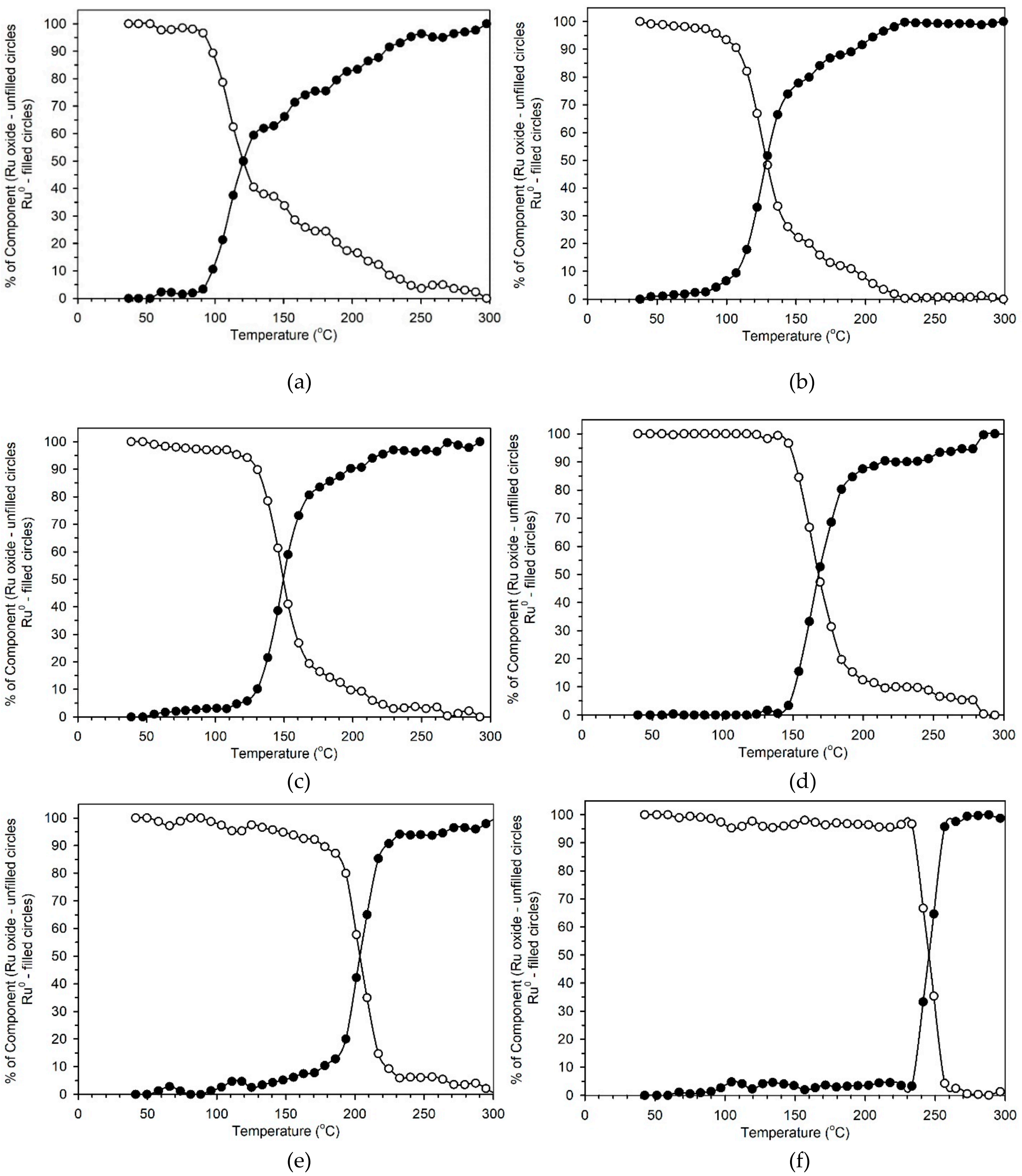

Figure S3: Linear combination fittings of TPR-XANES spectra recorded at the Ru K edge as a function of temperature for (a) unpromoted 1%Ru/m-zirconia catalyst and promoted catalysts with Na loadings of (b) 0.5%, (c) 1.0%, (d) 1.8%, (e) 2.5%, and (f) 5%, including (unfilled circles) ruthenium oxide and (filled circles) ruthenium metal. The point of 50% conversion from ruthenium oxide to ruthenium metal occurs at (a) 121°C, (b) 129°C, (c) 149°C, (d) 168°C, (e) 203°C, and (f) 246°C.

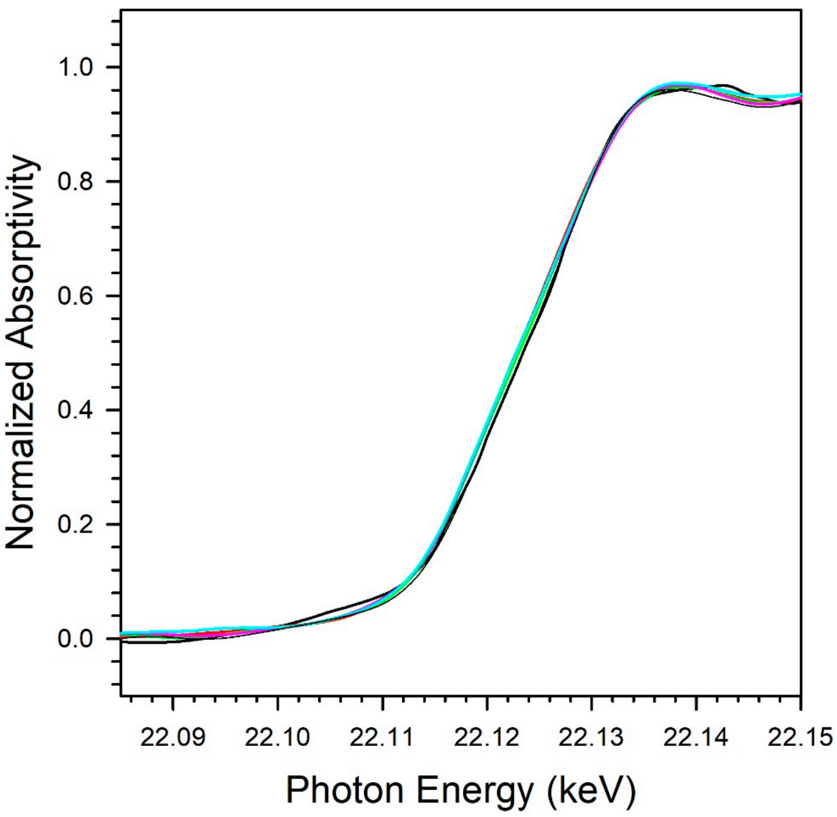

Figure S4: XANES overlays following H_2-TPR.

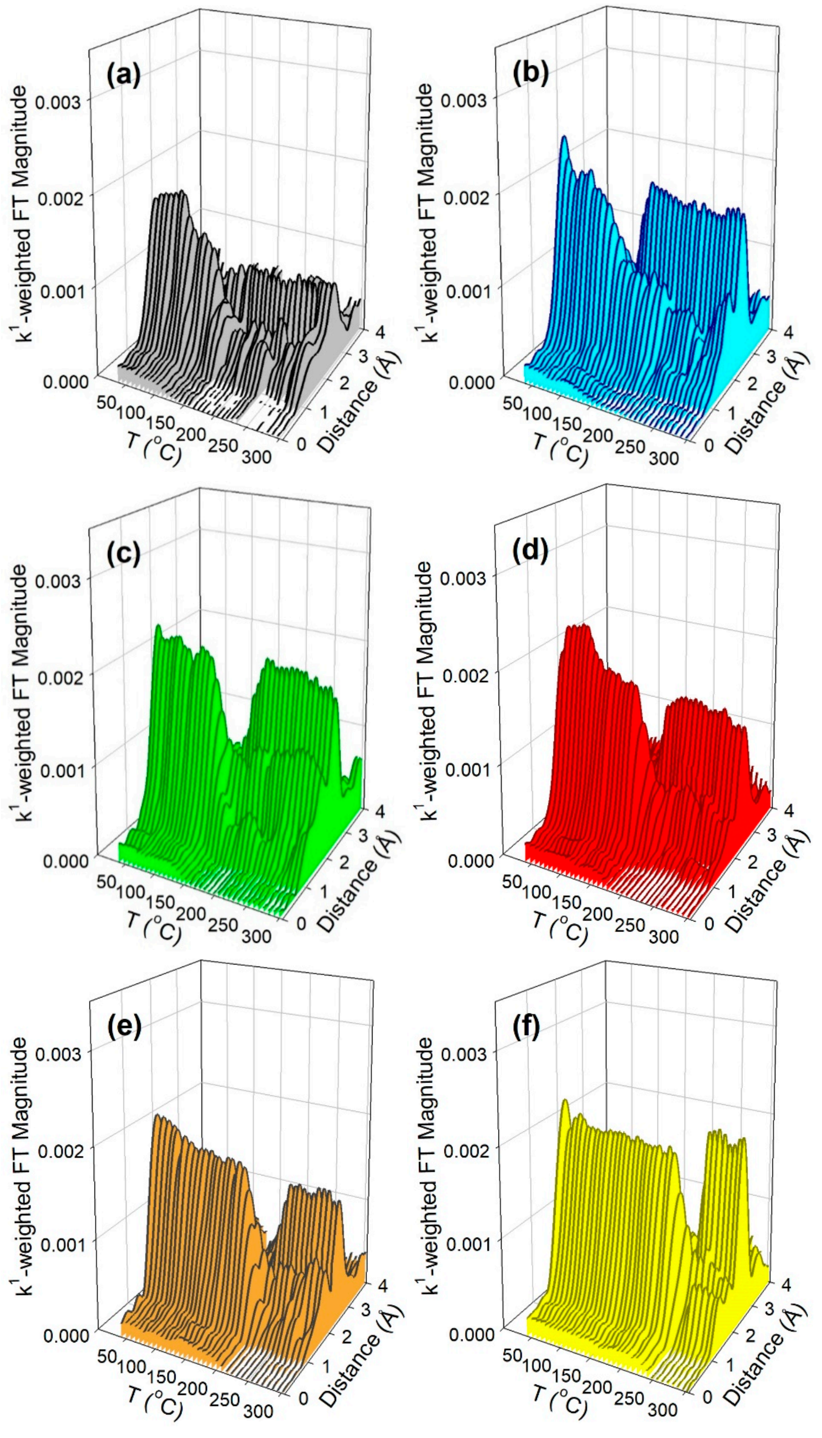

Figure S5: TPR-EXAFS Fourier transform magnitude spectra recorded near the Ru K edge as a function of temperature for (a, gray) unpromoted 1%Ru/m-zirconia and promoted catalysts having Na loadings of (b, cyan) 0.5%, (c, green) 1.0%, (d, red) 1.8%, (e, orange) 2.5%, and (f, yellow) 5%.

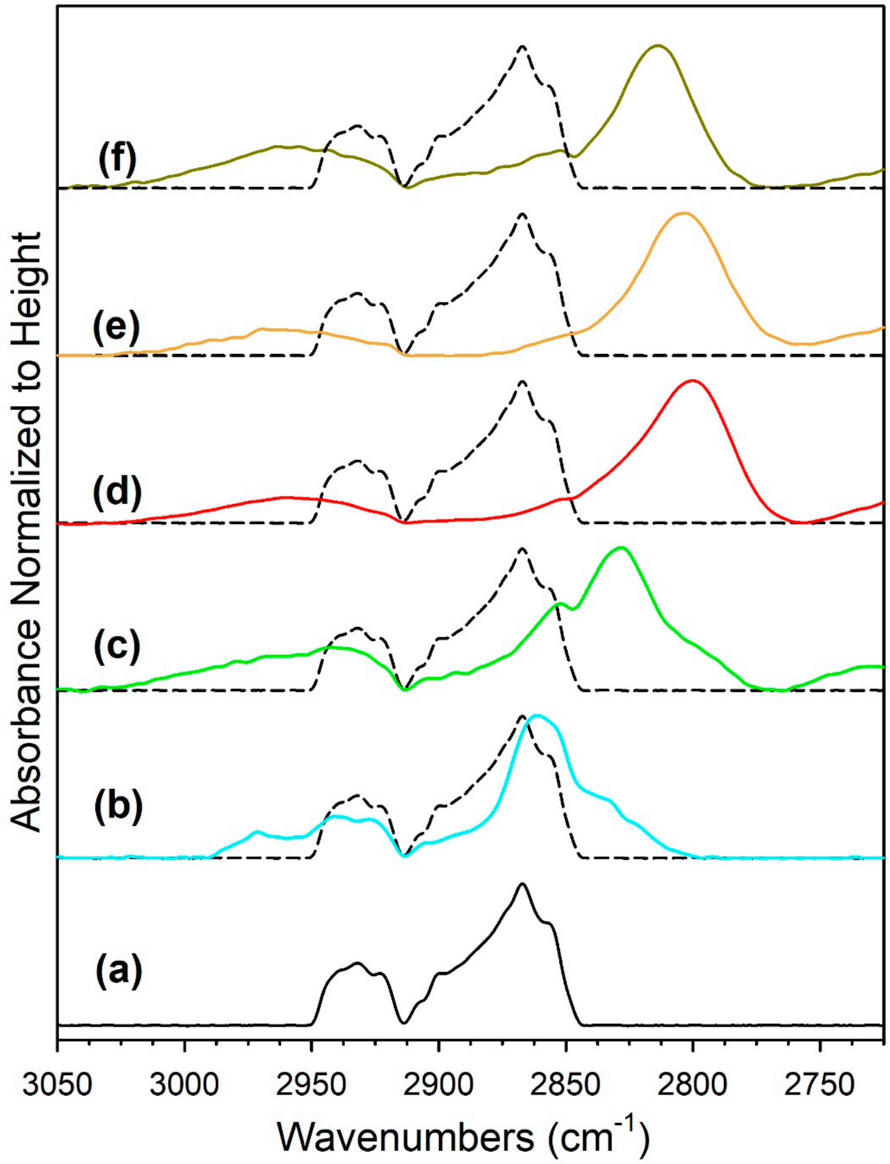

Figure S6: DRIFTS of formate ν(CH) band following CO adsorption at 225 °C (and cooling to 130 °C) to react with defect-associated OH groups produced over catalysts reduced at 300°C in H_2 and steamed at 225 °C (followed by H_2 and He purges at 225 °C), including (a) unpromoted 1%Ru/m-zirconia, and the promoted catalysts having Na loadings of (b) 0.5%, (c) 1%, (d) 1.8%, (e) 2.5%, and (f) 5%.

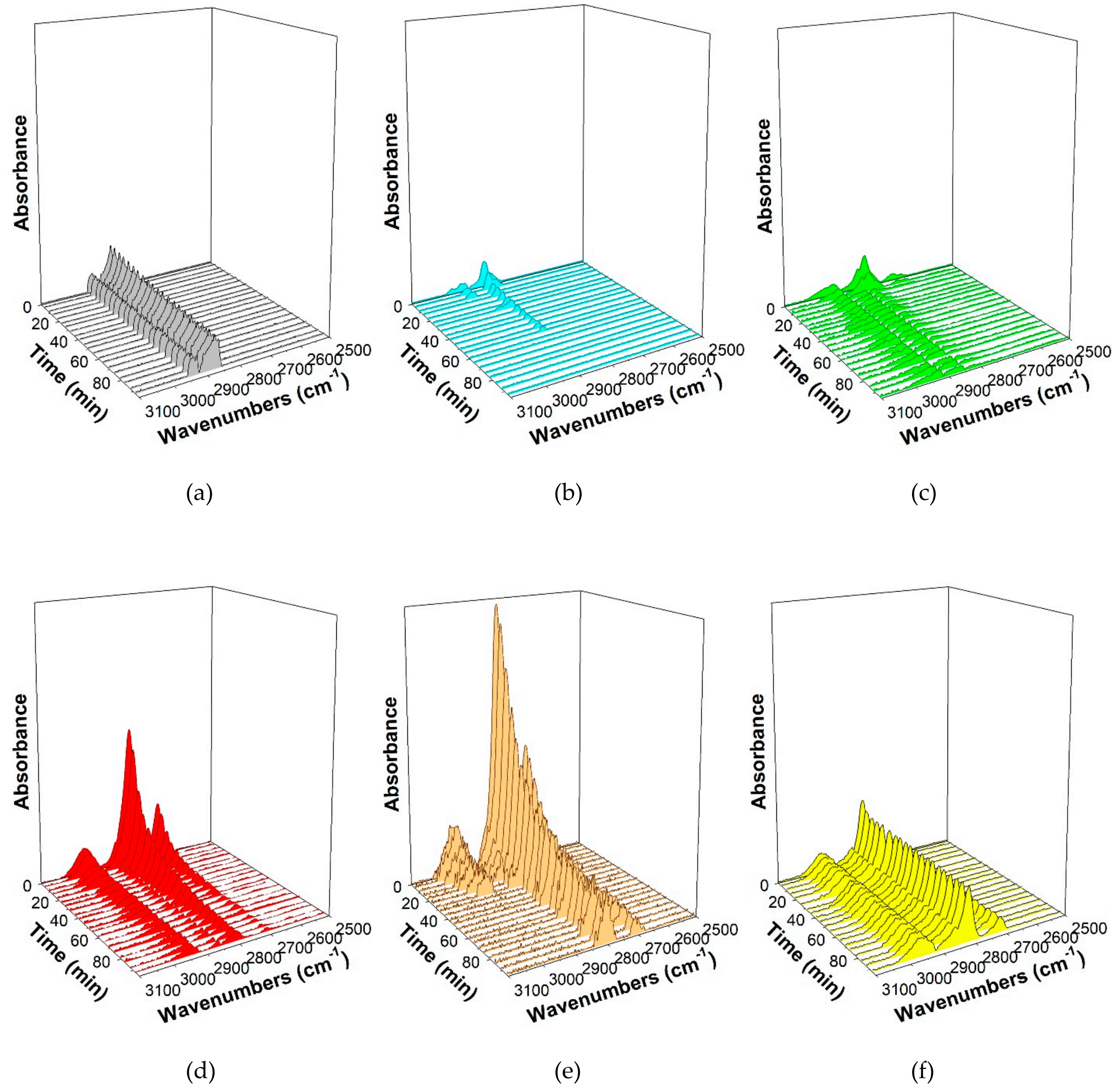

Figure S7: Forward formate decomposition in 75 mL/min of 4.4%H_2O in helium at 130°C for (a) unpromoted 1%Ru/m-zirconia, and the promoted catalysts having Na loadings of (b) 0.5%, (c) 1%, (d) 1.8%, (e) 2.5%, and (f) 5%.

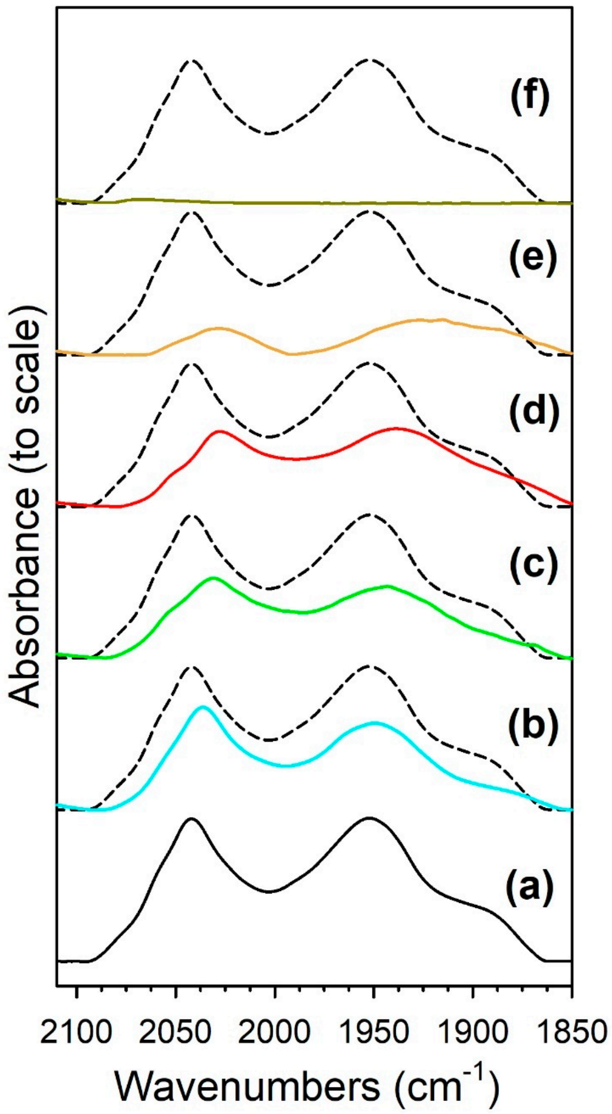

Figure S8: Effect of Na on attenuating the Ru carbonyl ν(CO) band intensity following CO adsorption at 225 °C (and cooling to 130 °C) over catalysts reduced at 300°C in H_2 and steamed at 225 °C (followed by H_2 and He purges at 225 °C), including (a) unpromoted 1%Ru/m-zirconia, and the promoted catalysts having Na loadings of (b) 0.5%, (c) 1%, (d) 1.8%, (e) 2.5%, and (f) 5%.

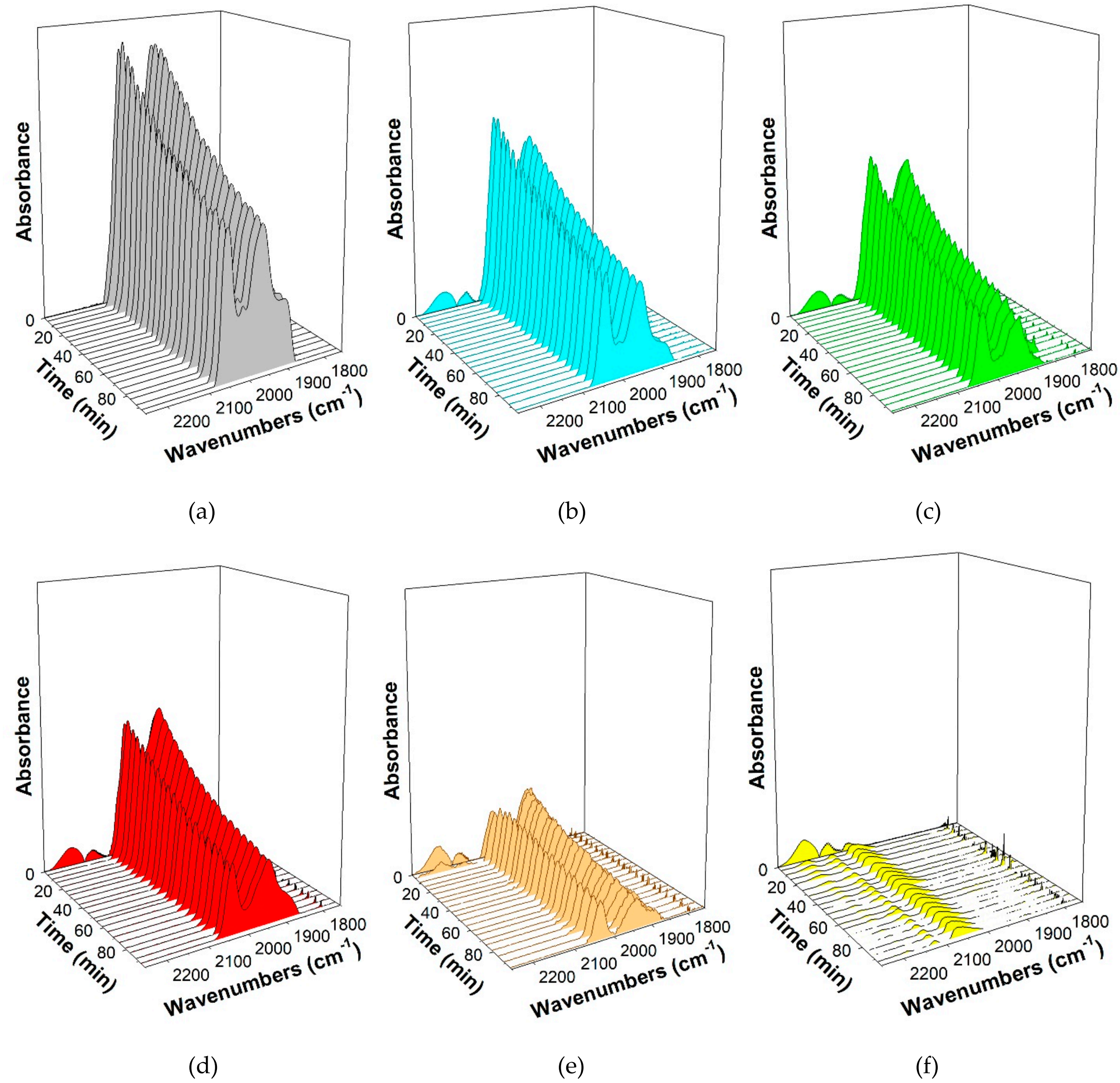

Figure S9: Ru-carbonyl decomposition in 75 mL/min of 4.4%H_2O in helium at 130°C for (a) unpromoted 1%Ru/m-zirconia, and the promoted catalysts having Na loadings of (b) 0.5%, (c) 1%, (d) 1.8%, (e) 2.5%, and (f) 5%.

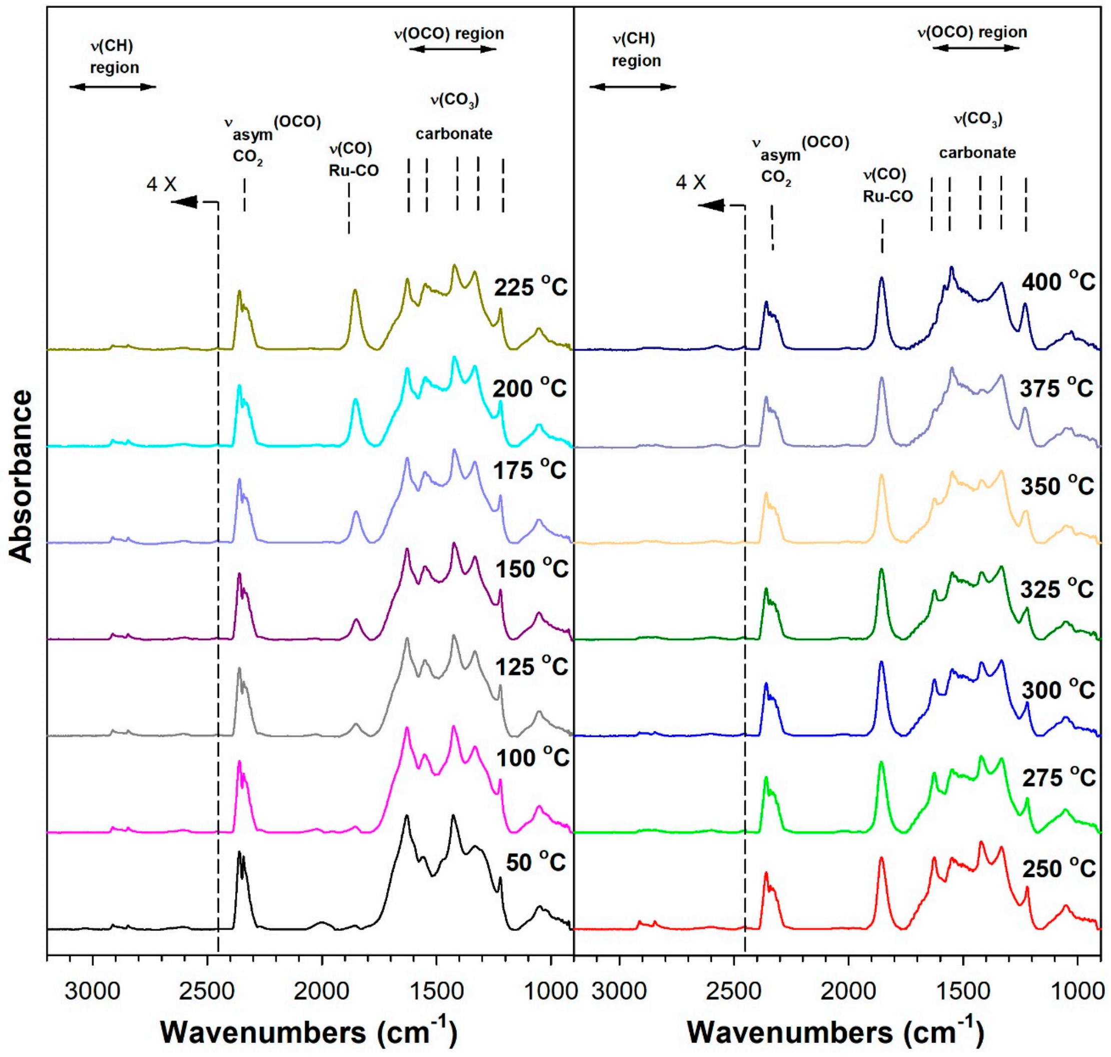

Figure S10: DRIFTS of 4%CO_2 (balance He) for unpromoted 1%Ru/m-zirconia reduced at 300°C in H_2, purged in He, and then cooled to 50 °C prior to CO_2 adsorption.

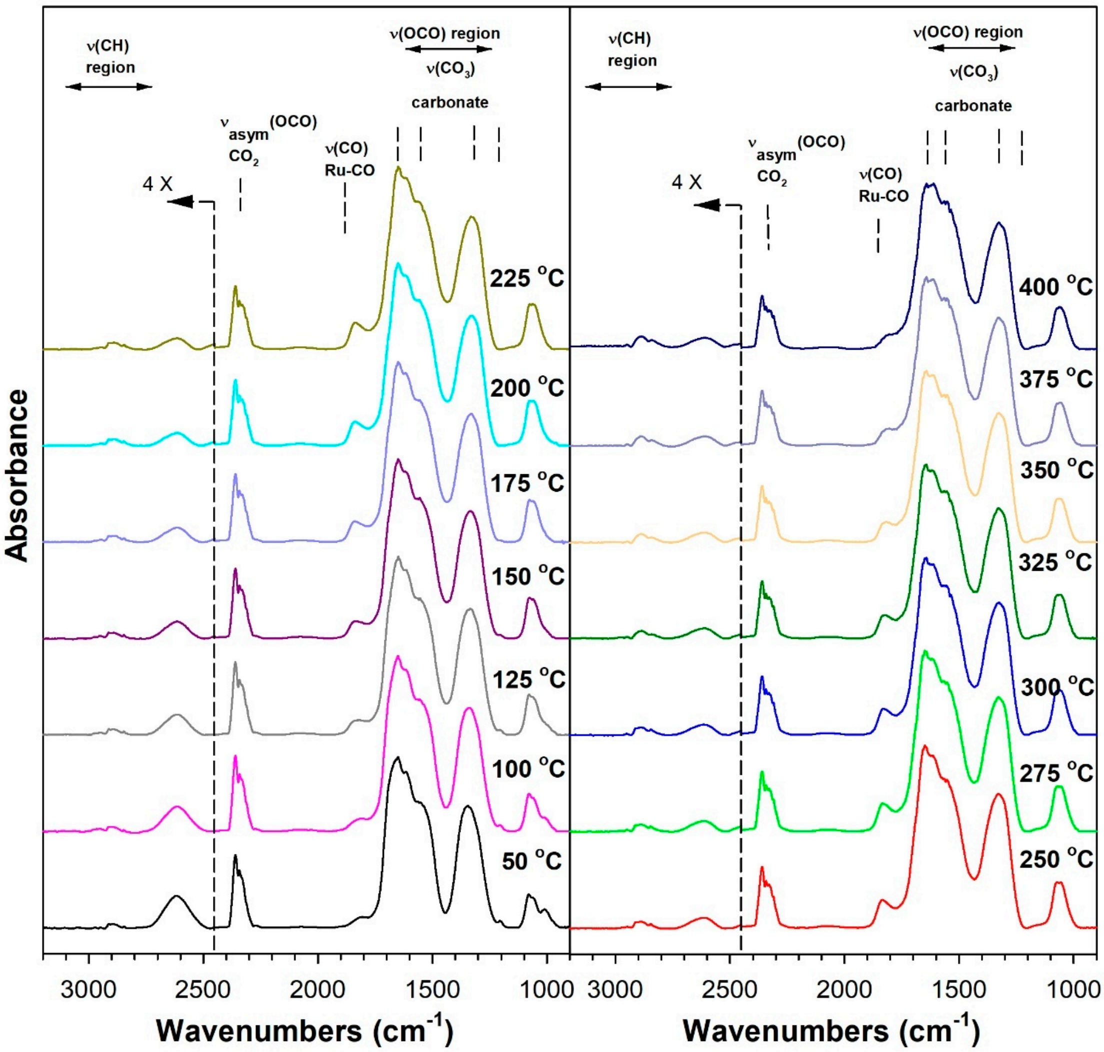

Figure S11: DRIFTS of 4%CO_2 (balance He) for 2.5%Na-1%Ru/m-zirconia reduced at 300°C in H_2, purged in He, and then cooled to 50 °C prior to CO_2 adsorption.
